# Machine learning-enhanced confocal Raman imaging enables label-free diagnosis and spatial metabolic profiling of isoniazid-induced hepatotoxicity

**DOI:** 10.7150/thno.119785

**Published:** 2025-08-30

**Authors:** Shimei Wang, Xiaoren Wang, Xudong Cui, Xiaotong Xie, Zhu Zhu, Tomii Ayaka, Renxing Song, Liping Zhou, Jin Sun, Li Zhang, Ruisheng Ge, Lei Yu, Yang Li

**Affiliations:** 1 Department of Infectious Disease, The Fourth Affiliated Hospital of Harbin Medical University, Harbin 150001, PR China.; 2 Research Center for Innovative Technology of Pharmaceutical Analysis, College of Pharmacy, Harbin Medical University, Harbin 150081, PR China.; 3 Department of Infectious Disease, The Second Affiliated Hospital of Harbin Medical University, Harbin 150001, PR China.; 4 Cardiovascular Center, Inner Mongolia People's Hospital, Hohhot 010010, PR China.; 5 State Key Laboratory of Frigid Zone Cardiovascular Diseases (SKLFZCD), College of Pharmacy, Harbin Medical University, Harbin 150081, PR China.; 6 Research Unit of Health Sciences and Technology (HST), Faculty of Medicine University of Oulu, Oulu 90220, Finland.; 7 Department of Pathology, The Fourth Affiliated Hospital of Harbin Medical University, Harbin 150001, PR China.

**Keywords:** isoniazid-induced liver injury, Raman spectroscopy imaging, machine learning, chemical composition, metabolomics

## Abstract

**Rationale:** Isoniazid-induced liver injury (INH-ILI) poses a significant clinical challenge due to the lack of reliable, non-invasive, and real-time diagnostic tools. Here, we present an integrated platform that combines label-free confocal Raman spectroscopy imaging, machine learning (ML), and targeted metabolomics to identify and classify INH-ILI in a murine model.

**Methods:** An INH-ILI mouse model was established, and Raman imaging and subsequent data analysis were performed on the control and INH-ILI at 7, 14, 21, and 28-day groups. Alterations in hepatic metabolites following INH-ILI were elucidated. Furthermore, ML techniques were employed to identify subtle differences between the control and INH-ILI groups.

**Results:** Distinct Raman spectral shifts, notably the emergence of a 1638 cm^-1^ peak in injured liver tissues compared to characteristic peaks at 1203, 1266, and 1746 cm^-1^ in controls, were observed. ML models including support vector machine (SVM), random forest (RF), extreme gradient boosting (XGBoost), and convolutional neural network (CNN) have achieved accurate staging and classification of INH-ILI (AUC > 0.95). Metabolomic analysis further confirmed disruptions in lipid and aromatic amino acid metabolism, particularly involving phenylalanine-tyrosine imbalance linked to oxidative stress.

**Conclusions:** This method enables precise, high-throughput, and spatially resolved diagnosis of INH-ILI, with strong potential for clinical translation in drug-induced liver injury assessment.

## Introduction

Tuberculosis (TB) remains one of the most pressing global health challenges, ranking as the second leading cause of death from infectious diseases worldwide in 2022, surpassed only by COVID-19 1-3. Despite decades of public health efforts, TB continues to infect millions and exerts a profound burden on patients, families, and healthcare systems. Isoniazid (INH), a first-line anti-tuberculosis agent, plays a critical role in TB treatment due to its high efficacy, affordability, and broad use in both active and latent TB cases. However, INH is also one of the most frequent causes of drug-induced liver injury (DILI), leading to a spectrum of hepatic manifestations ranging from asymptomatic enzyme elevation to acute liver failure and death 4-6. INH-induced liver injury (INH-ILI) has been reported to occur in up to 20% of patients and is a major reason for treatment interruption and poor clinical outcomes.

Clinically, the diagnosis of INH-ILI remains challenging. Liver biopsy, though considered the gold standard, is invasive, associated with procedural risks, and often suffers from sampling variability and inter-observer inconsistencies. The pathological features of INH-ILI are heterogeneous, involving hepatocytes, bile ducts, and vascular endothelium in varying degrees 7, 8. Moreover, access to experienced pathologists and histopathology services is uneven, especially in resource-limited settings where TB burden is highest. Serum biomarkers such as alanine aminotransferase (ALT) and aspartate aminotransferase (AST) are routinely used but lack specificity and spatial resolution. These limitations highlight the urgent need for a non-invasive, accurate, and dynamic method for identifying INH-ILI and monitoring its progression.

Raman spectroscopy, a label-free and non-destructive optical technique based on inelastic light scattering by molecular vibrations, has emerged as a powerful analytical tool in biomedicine 9-12. It offers molecular “fingerprints” of biological samples, enabling the detection of subtle biochemical changes associated with disease processes. In recent years, Raman spectroscopy has shown promise in the diagnosis of various cancers, infections, and metabolic disorders, including applications in tissue analysis, biofluid screening, and intraoperative margin assessment 13-15. However, its application in detecting hepatotoxicity, particularly INH-ILI, remains underexplored.

A critical barrier to the clinical translation of Raman spectroscopy lies in the complexity of spectral data, which necessitates labor-intensive expert interpretation prone to subjectivity. To overcome this, we developed an integrated diagnostic platform combining label-free confocal Raman imaging, machine learning (ML)-driven spectral decoding, and targeted metabolomic validation. This synergy enables automated recognition of disease-specific molecular fingerprints, achieving rapid, operator-independent classification of tissue pathology while simultaneously providing spatially resolved metabolic insights 16-19. Such capabilities address the unmet clinical need for non-invasive, real-time assessment of DILI.

**Scheme 1:**
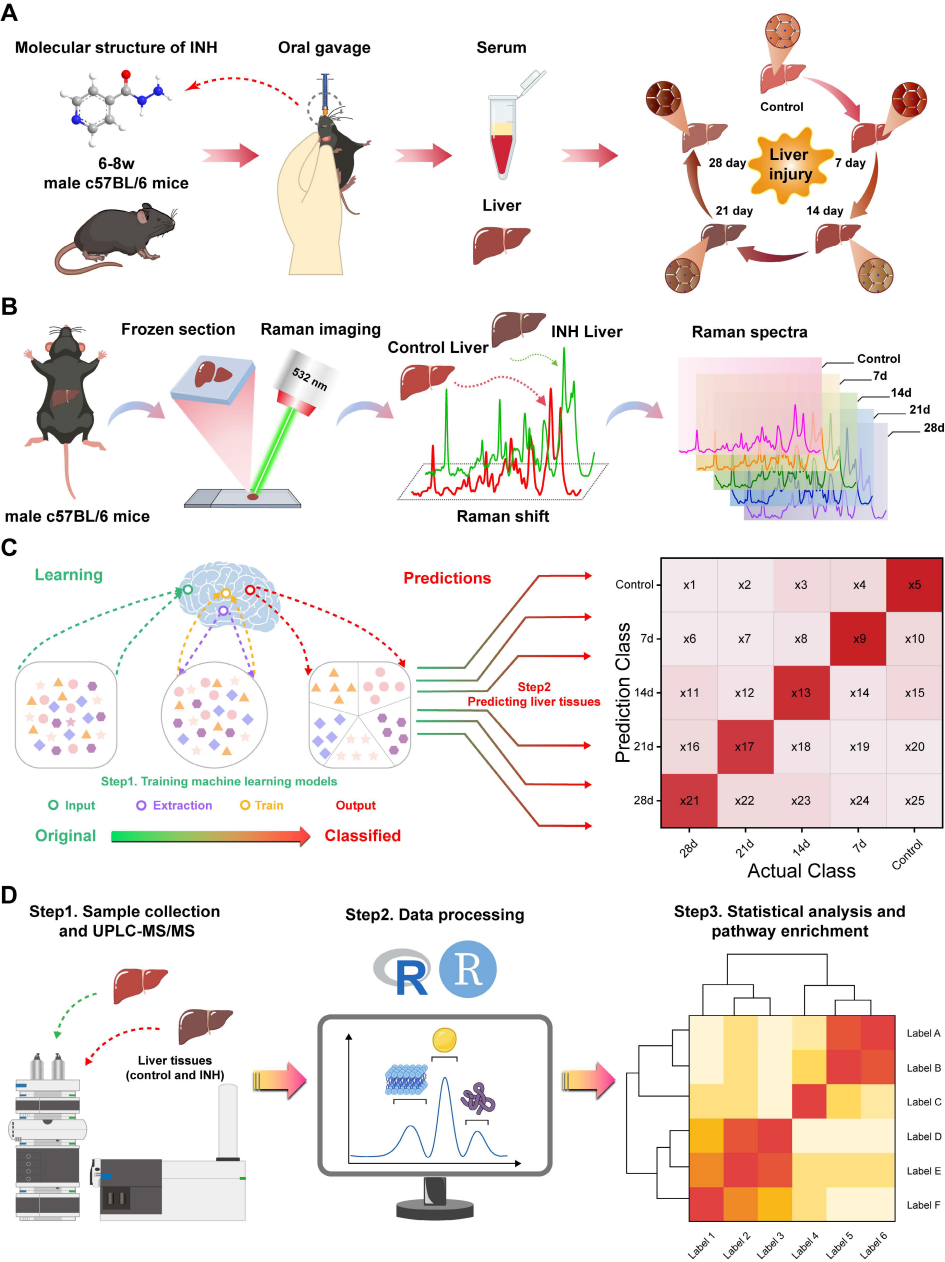
** Workflow for the metabolic component of INH-ILI liver tissue.** (**A**) Construction of the INH-ILI model in c57BL/CJ mice. (**B**) Flowchart illustrating Raman scattering imaging of liver tissue. (**C**) Overview of the ML analysis process. (**D**) Validation of Raman scattering imaging analysis results through widely targeted metabolomics utilizing UPLC-MS/MS.

In this study, we propose a novel diagnostic framework that integrates confocal Raman spectroscopy imaging with ML and targeted metabolomics to identify INH-ILI in a murine model. We constructed a time-course mouse model of INH-ILI and performed label-free Raman imaging of liver tissues at different stages of injury. Using ML algorithms, we classified liver injury stages with high accuracy. Furthermore, we validated spectral findings through metabolomics, revealing key alterations in lipid and aromatic amino acid metabolism. This integrative approach offers a non-invasive, spatially resolved, and molecularly specific method for identifying INH-ILI and lays a foundation for future clinical translation in DILI diagnosis and mechanistic studies, as shown in Scheme 1.

## Experimental Section

### Reagents

INH (analytical standard, ≥ 99%, lot number I3377) and tribromoethanol (≥ 97%, lot number T48402) were procured from Sigma Aldrich Co., Ltd. (Shanghai, China), 4% paraformaldehyde solution (lot number BL539A) was obtained from Biosharp Biotechnology (Hefei, China), optimal cutting temperature compound (OCT, lot number 4583) was acquired from Sakura Finetek USA, Inc. (Torrance, USA). Colorimetric assay kits for ALT and AST were purchased from Elabscience Biotechnology Co. Ltd. (Wuhan, China).

### Animal Model

**Figure 1:**
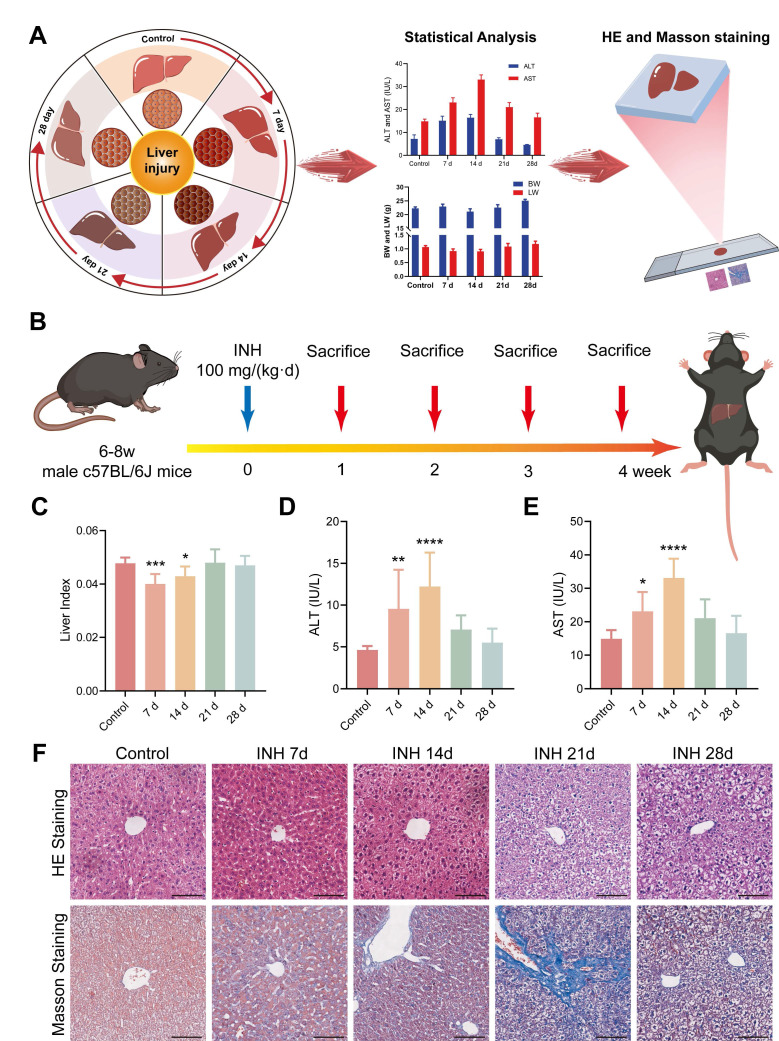
**Serologic and histopathologic characteristics of mice in the control and INH-ILI groups.** (**A**) Schematic representation detailing serological and histopathological characteristics observed in mice. (**B**) Methodology for establishing the INH-ILI mouse model. (**C**) Comparison of liver index between control and INH-ILI groups, respectively (n = 10). Alterations in serum ALT (**D**) and AST (**E**) levels across control and INH-ILI groups at various time points are presented accordingly (n = 10). (**F**) Representative HE staining alongside Masson's staining is conducted on liver sections from control and INH-ILI groups, respectively. Magnification, ×100. All scale bars are 100 μm. All the Data are presented as mean ± SD. *P < 0.05, **P < 0.01, ***P < 0.001, ****P < 0.0001 versus the control group.

Previous research has shown that mice are more suitable for developing animal models of INH-ILI that closely resemble human physiology 20. C57BL/6J mice (male, 6-8 weeks, 18-22g, Liaoning Changsheng Biotechnology Co., Ltd.) were housed under standard pathogen-free (SPF) conditions at 24 ± 2 ℃, 12 h light/dark cycle, and 50 ± 5% relative humidity. After a one-week adaptive feeding, 50 mice were randomly assigned to a control group (*n* = 10) or an experimental group (*n* = 40). The experimental group was subdivided into four subgroups according to the treatment duration: INH treatment for 7, 14, 21, and 28 days (*n* = 10 in each group). Mice in the experimental group received INH via intragastric gavage at a dose of 100 mg/kg once daily 21-23. In contrast, those in the control group received an equivalent volume of purified water according to body weight. Figure 1B shows the specific procedural steps involved. After the final administration, the mice were fasted for 24 h before being anesthetized with tribromoethanol. Blood samples were collected via orbital vein puncture and subsequently centrifuged at 4 ℃ and 3500 rpm for 15 min to obtain serum, then frozen at -80 ℃ for subsequent biochemical detection. The liver tissue was taken and weighed after the mice were killed by neck removal. The liver index was calculated as (liver weight/body weight) × 100. The liver tissue was divided into three segments: one segment was fixed in a solution of 4% paraformaldehyde for 24 h before embedding in paraffin, another segment was frozen in OCT for Raman scattering imaging, and the last segment was preserved at -80 °C for subsequent experiments. All animal experiments were approved by the Institutional Animal Care and Use Committees of the Harbin Medical University (ethics code: IRB3072724).

### Biochemical Assay and Histological Analysis

Serum levels of ALT and AST were quantified using colorimetric assay kits following all procedures outlined by the manufacturer's instructions. Paraffin-embedded liver sections (5 μm thick) were stained with hematoxylin-eosin (HE) and Masson, respectively. Images were captured using a DMI3000B fluorescence microscope (Leica, Wetzlar, Germany).

### Raman Scattering Imaging of Liver Tissue

For Raman measurements, 10 μm frozen liver tissue sections were prepared and mounted on glass slides. Raman spectroscopy and imaging of liver tissue were conducted using a WITec Alpha 300R Raman instrument (Ulm, Germany). A laser with a wavelength of 532 nm, objective lens of L × 100 (numerical aperture (NA) = 0.9, working distance (WD) = 1 mm), laser power of 12.5 mW per data point, and exposure time of 0.35 s were selected. Spectra were collected from a minimum of 100 randomly chosen points on the surface of each tissue sample within the range of 600-1800 cm^-1^. The Raman imaging area of the liver tissue was 80 × 80 μm; the other conditions were the same as before, and the temperature of the above operating experiment was always maintained at 24 ℃.

Before statistical analysis, spectral data were processed using the WITec Project (version 5.3, Ulm, Germany) and LabSpec software (version 5.0, Horiba, Japan), including smoothing, baseline reduction, cosmic ray removal, fluorescent background removal, and signal-to-noise ratio improvement. Origin 2024 was used to compute the mean and standard deviation (SD) for each spectra group.

### Machine Learning

Spectra of 30 Raman shifts (from 600 to 1800 cm^-1^) were obtained as uniform manifold approximation and projection (UMAP) and t-distributed stochastic neighbor embedding (t-SNE) in each of the five groups of spectra. The Raman spectra were mapped onto a score map using the "error ellipse" function in UMAP and tSNE to draw an error ellipse with a confidence of 95%. The support vector machines (SVM), random forest (RF), extreme gradient boosting (XGBoost), and convolutional neural network (CNN) methods were adopted to conduct spectral analyses of 1000 Raman shifts within the range of 600-1800 cm^-1^ for five groups of spectra respectively. The area under the curve (AUC) of the receiver operating characteristic (ROC) curve was used to classify identification performance. The above spectral data were all analyzed in the R Studio 4.3.0 environment.

### UPLC-MS/MS Metabolomics and Data Analysis

Based on the above findings, collected six liver tissue samples from the control and INH 7-, 14-, and 28-day groups for this investigation. The data acquisition system primarily consisted of electrospray ionization (ESI), tandem mass spectrometry (MS/MS, QTRAP®6500+ System), and ultra-performance liquid chromatography (UPLC, ExionLC AD).

R Studio 4.3.0 software was used to perform principal component analysis (PCA), partial least squares-discriminant analysis (PLS-DA), orthogonal partial least discriminant analysis (OPLS-DA), and the Kyoto Encyclopedia of Genes and Genomes (KEGG) pathway database. The data were annotated using the KEGG Compound database, which widely targeted metabolomics data.

### Statistical Analysis

Data are presented as the mean ± SD. Student *t*-test was used for comparisons between the two groups. One-way analysis of variance (ANOVA) was used for multiple comparisons. GraphPad Prism software (version 9.5, San Diego, CA, USA) was used for statistical analysis. *P* < 0.05 is regarded as statistically significant (*P < 0.05, **P < 0.01, ***P < 0.001, ****P < 0.0001).

## Results and Discussion

### Establishment and Validation of the INH-ILI Mouse Model

To validate the INH-ILI model, both serological biomarkers and histopathological changes were assessed at multiple time points (Figure 1A). Mice treated with INH exhibited significant alterations in liver index, body weight (BW), and liver weight (LW) compared to the control group. These differences were evident as early as days 7 and 14 (Figure 1C), with further disparities observed at later time points (Figure S1A and S1B). In particular, serum levels of ALT and AST were markedly elevated following INH administration, peaking at day 14 (Figure 1D-E), indicating hepatocellular damage.

Histopathological evaluation of hematoxylin and eosin (H&E)-stained sections revealed progressive hepatic injury in the INH-treated groups, with varying degrees of hepatocyte swelling, cytoplasmic vacuolization, and necrosis observed on days 7, 14, 21, and 28 (Figure 1F). Notably, the severity of liver injury increased over time, reaching its peak at day 21 before partially subsiding by day 28.

Furthermore, Masson's trichrome staining showed increased collagen fiber deposition, particularly at day 21, indicating hepatic fibrosis as a consequence of sustained hepatocellular injury (Figure 1F). These combined serological and histological findings confirm the successful establishment of an INH-ILI model that captures the temporal progression of liver injury, suitable for subsequent Raman-based molecular analysis.

### Confocal Raman Imaging Reveals Molecular Signatures of INH-ILI

**Figure 2:**
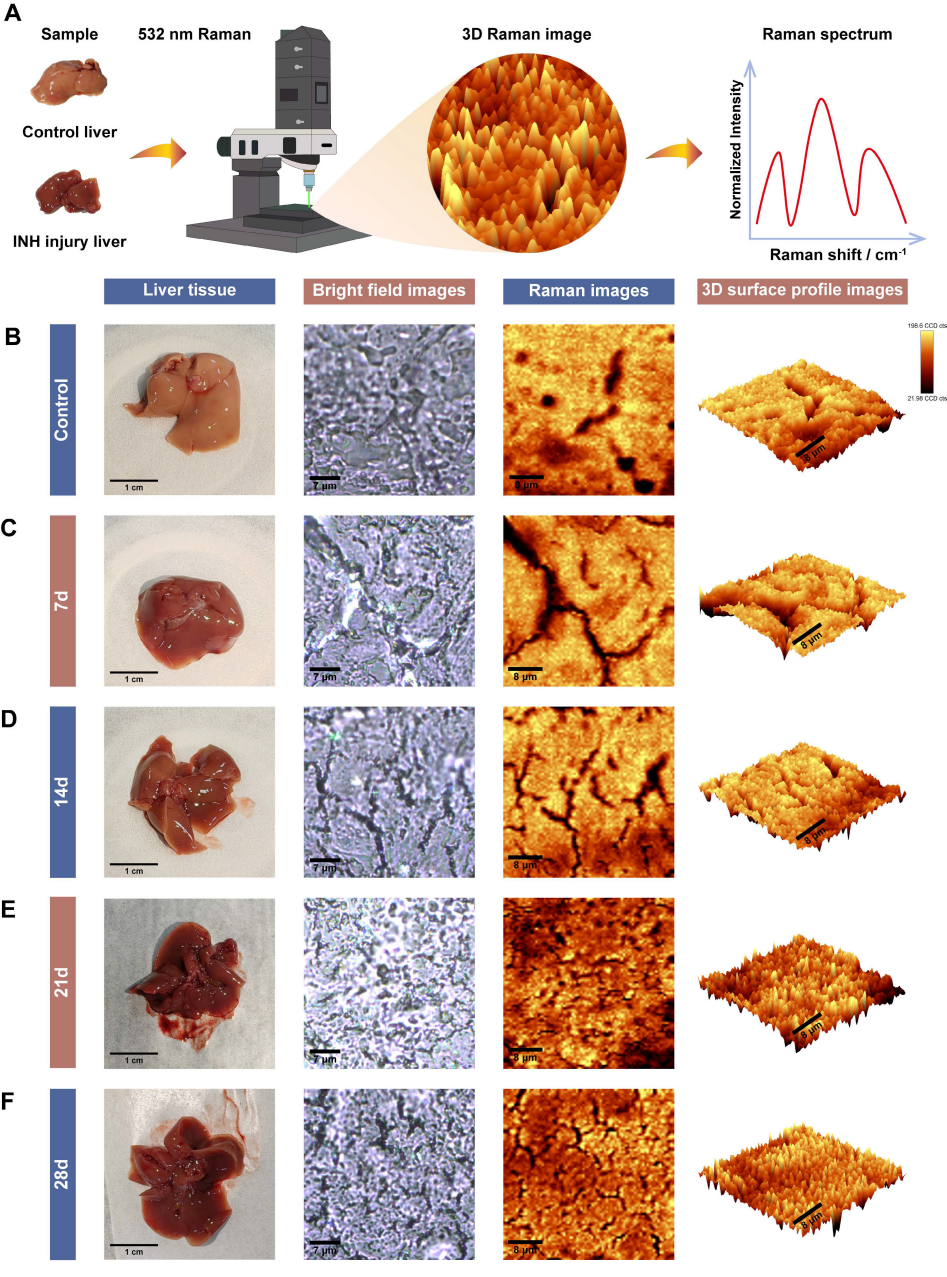
**Raman scattering imaging of control and INH-ILI groups.** (**A**) Schematic representation depicting the methodology employed for Raman scattering imaging. (**B**-**F**) Representative comparative images representing liver tissues, bright field images, Raman images, and 3D surface profile images for the control and INH-ILI groups.

To explore the molecular changes associated with INH-ILI, label-free confocal Raman scattering imaging was performed using a 532 nm excitation laser. The workflow of spectral acquisition and analysis is illustrated in Figure 2A. Frozen liver sections from the control and INH-treated mice at 7, 14, 21, and 28 days were imaged. Representative results are shown in Figure 2B-F, including bright-field images, Raman intensity maps, and corresponding three-dimensional surface plots of the scanned areas.

The Raman signals obtained from liver tissues were of high quality and reproducibility across groups. Notably, no enhancement substrates or nanoparticles were employed, avoiding potential signal interference or biological risks associated with nanomaterials. This substrate-free approach not only simplifies sample preparation but also enhances safety and feasibility for broader clinical or translational applications, particularly in resource-limited settings 24, 25. Material-free confocal Raman imaging thus offers a promising route for visualizing biochemical changes in liver tissue with high spatial resolution. The robust spectral signals obtained provide a molecular basis for further classification of healthy and diseased tissues, forming the foundation for the integration of artificial intelligence algorithms in the subsequent analysis.

### Molecular Analysis of Liver Tissue Using Raman Spectroscopy

To investigate the biochemical alterations associated with INH-ILI, high-resolution Raman spectral data were collected from frozen liver tissue sections (Figure S2). At least 1,000 spectra were randomly acquired per sample across each group to ensure representative and statistically robust profiling.

**Figure 3:**
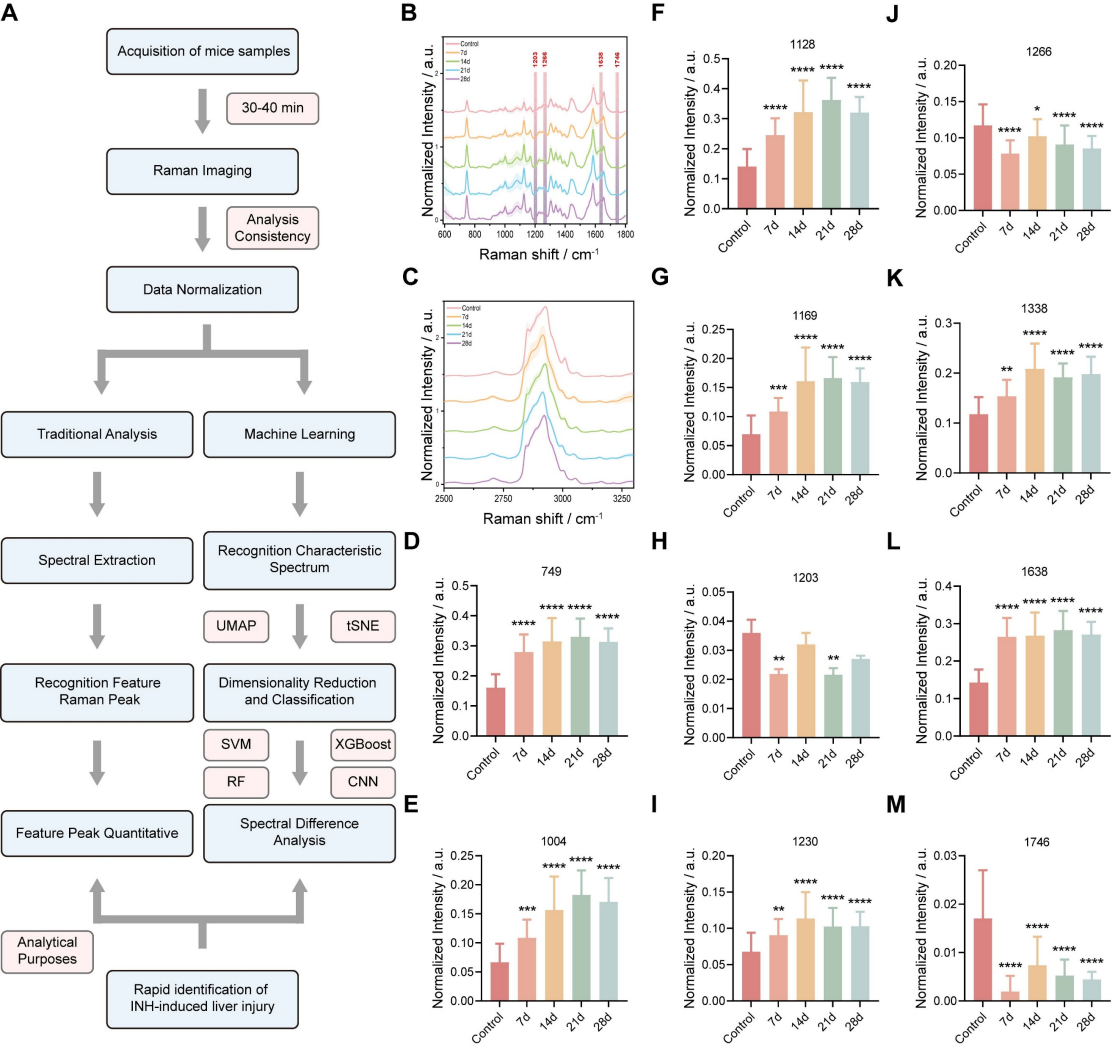
** Raman spectral difference in characteristic peak intensity between control and INH-ILI groups.** (**A**) Flowchart illustrating the processing of Raman spectra. The average Raman spectra in the ranges of 600-1800 cm^-1^ (**B**) and 2500-3300 cm^-1^ (**C**) were obtained from the control and INH-ILI groups, respectively. The central lines represent the mean values, while the shaded areas indicate standard deviations of these means. A gray dotted line marks a characteristic peak distinguishing between the control and INH-ILI groups. Peak intensities were measured at (**D**) 749, (**E**) 1004, (**F**) 1128, (**G**) 1169, (**H**) 1203, (**I**) 1230, (**J**) 1266, (**K**) 1338, (**L**) 1638, and (**M**) 1746 cm^-1^ for both control and INH-ILI groups, respectively. All the data are presented as mean ± SD. *P < 0.05, **P < 0.01, ***P < 0.001, ****P < 0.0001 versus the control group.

Seventeen and fifteen distinct Raman peaks were consistently observed in control and INH-ILI tissues, respectively. Figure 3A presents a flowchart of the Raman spectrum processing. These peaks corresponded to major molecular classes, including lipids, amino acids, proteins, and nucleic acids. Specifically, peaks at 1128, 1266, 1366, 1441, 1657, and 1746 cm^-1^ were predominantly lipid-associated. Peaks at 749, 1004, 1169, 1203, 1395, and 1589 cm^-1^ were linked to aromatic amino acids such as tyrosine, tryptophan, and phenylalanine. The Raman line at 1230 cm^-1^ represents the antisymmetric phosphate stretching of the amide III band (β-pleated sheet). The Raman signature at 1082 cm^-1^ corresponds to the symmetric stretching of PO- 2 in nucleic acids. The Raman feature at 970 cm^-1^ is attributed to the phosphate monoester groups in the phosphorylated protein. The peaks at 1306 and 1338 cm^-1^ were assigned to CH_3_/CH_2_ twisting or bending and CH_2_ deformation of collagen, respectively. The band at 1638 cm^-1^ represents the intermolecular bending mode of water.

The difference in Raman spectra between the control and INH-ILI groups reflects alterations in the biochemical components of the liver tissue caused by INH, providing a basis for distinguishing between control and INH-ILI liver tissues. Table S1 presents the leading positions of the Raman vibration peaks and the corresponding representative compounds reported in the literature 26.

To better identify the differences, the average Raman spectra of the control and INH-ILI groups were plotted in the range of 600-1800 cm^-1^ (Figure 3B) and 2500-3300 cm^-1^ (Figure 3C), respectively, and heat maps were constructed (Figure S4A-B). In addition, Figure 3B demonstrates the difference in the intensity of the Raman characteristic peaks between the control and INH-ILI groups within the 600-1800 cm^-1^ range.

To further analyzed and examined the differences in the metabolic components of the liver tissues between the control and INH-ILI groups based on the changes in the Raman characteristic peaks. Most of the aromatic amino acids, amides, collagen, and water-related peaks, such as 749 (Figure 3D), 1004 (Figure 3E), 1128 (Figure 3F), 1169 (Figure 3G), 1203 (Figure 3H), 1230 (Figure 3I), 1338 (Figure 3K), and 1638 cm^-1^ (Figure 3L), were higher in the INH-ILI groups (*P* < 0.05). In contrast, some lipid peaks, such as 1266 (Figure 3J) and 1746 cm^-1^ (Figure 3M), were lower in the INH-ILI groups (*P* < 0.05). Research has indicated that the liver is crucial in drug detoxification, including steatosis and phospholipidosis 27-29. This may serve as one of the critical pathological foundations for the occurrence and progression of INH-ILI. Peak intensities in the I INH-ILI 7-, 14-, 21-, and 28-day groups at wavelengths of 749 (Figure 3D), 1004 (Figure 3E),1128 (Figure 3F),1169 (Figure 3G),1230 (Figure 3I), and1338 cm^-1^ (Figure 3K) are found to be higher than those observed in the control group. Additionally, the peak intensity at wavelength 1366 (Figure S5D) cm^-1^ was elevated in the INH-ILI 14-, 21-, and 28-day groups compared to that in the control. After treatment with INH-ILI for 7 and 21 days, the peak intensity corresponding to the liver tissue at 1203 cm^-^¹ (Figure 3H) exceeded that recorded for the control group.

Interestingly, 1203,1266, and 1746 cm^-1^ were the characteristic peaks of the control group, while 1638 cm^-1^ was the characteristic peak distinctive of the INH-ILI groups (Figure 3L). 1203 cm^-1^ linked to aromatic amino acids (phenylalanine/tryptophan), 1266 cm^-1^ assigned to amide III (β-sheet proteins) and lipids, and 1746 cm^-1^ characteristic of C=O ester bonds in lipids. These peaks decreased in INH-ILI tissues, consistent with disrupted lipid/protein homeostasis. Control-associated peaks (1203, 1266, and 1746 cm^-1^) declined progressively with injury duration, reflecting metabolic dysfunction rather than acute drug deposition. The peak at 1638 cm^-1^ is explicitly attributed to the intermolecular bending mode of water. This aligns with histopathological evidence of hepatocyte swelling, cytoplasmic vacuolization, and edema in INH-ILI tissues. 1638 cm^-1^ intensity peaked at day 21, mirroring maximal histopathological injury (hepatocyte necrosis/fibrosis; Figure 1F). It is worth noting that, the specificity of the 1638 cm^-1^ peak for INH-ILI-induced liver injury arises not from water itself, but from pathology-driven alterations in the aqueous microenvironment. In INH-ILI, cellular damage releases macromolecules (proteins, nucleic acids, lipids) and disrupts ion homeostasis. Increased bound water populations near hydrophilic macromolecule surfaces, where water molecules exhibit restricted mobility and altered hydrogen-bonding networks. Modify local ionic strength (e.g., K^+^/Ca^2+^ leakage), perturbing water structure through ion hydration effects. The 1638 cm^-1^ bending mode is highly sensitive to such constrained hydrogen-bonding environments. Thus, its increase in INH-ILI reflects liver-specific pathophysiological restructuring of water, not generic hydration changes. In our experiment, only the OH-stretch in the 7-day INH-ILI group (with the center at approximately 3250-3350 cm^-1^) originated from strong hydrogen bond water, while the shoulders at 3400-3500 cm^-1^ reflected weaker hydrogen bonds (Figure S3). In addition, the OH-stretching region is susceptible to fluorescence interference in biological tissues. It indicates that the change of this bending mode has a relatively small correlation with the wider OH- stretching region.

**Figure 4:**
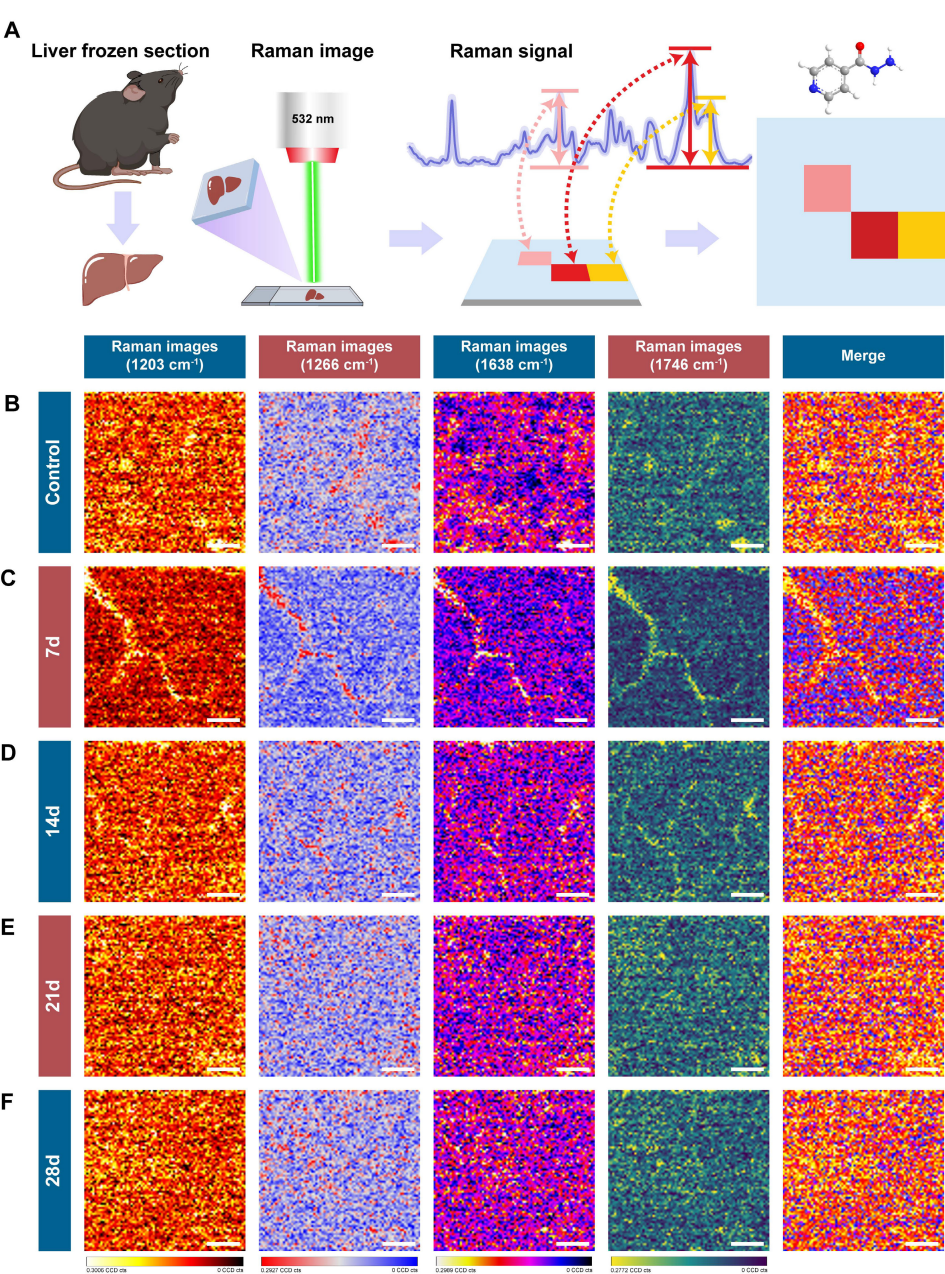
**Raman scattering imaging reveals the characteristic peaks of control versus INH-ILI groups.** (**A**) Schematic representation illustrating the methodology utilized for Raman scattering imaging of characteristic peaks. (**B**-**F**) Representative contrast images of characteristic peaks at 1203, 1266, 1638, and 1746 cm^-1^ of the control and INH-ILI groups and merge images of the above four images. All scale bars are 8 μm.

Figure S5 shows the signal intensities of other Raman peaks in the control and INH-ILI groups. The peak intensities at 970 (Figure S5A), 1082 (Figure S5B), 1306 (Figure S5C), 1395 (Figure S5E), 1441 (Figure S5F), and 1589 cm^-1^ (Figure S5G) were significantly higher in the INH-ILI 14-, 21-, and 28-day groups, respectively. Additionally, after 21 days of INH treatment, the peak intensity observed in the liver tissue at 1657 cm^-1^ (Figure S5H) exceeded that of the control group. Figure 4A illustrates a flowchart depicting the characteristic peaks of Raman scattering imaging for both the control and INH-ILI groups. Representative Raman scattering images corresponding to the characteristic peaks at 1203, 1266, 1638, and 1746 cm^-1^ were acquired for each group and are presented in distinct colors (Figure 4B-E). Identified the changes in the liver tissue metabolites based on the signal intensity of the two sets of Raman characteristic peaks. Together, these spectral analyses provided a reliable foundation for classifying healthy versus INH-injured tissue and demonstrated the capability of Raman spectroscopy to detect subtle biochemical transitions during hepatotoxic progression. The current Raman images were designed to provide preliminary spatial distribution data of target molecules, as the technique's primary strength lies in its label-free chemical mapping capability. While we acknowledge the need for deeper biological interpretation, the current dataset does not yet include paired histopathology or single-cell resolution data to correlate Raman patterns with cellular heterogeneity or disease states. Future research that combines Raman imaging with histology or spatial transcriptomics.

### Machine Learning Enables Precise Classification of Liver Injury Stages

**Figure 5:**
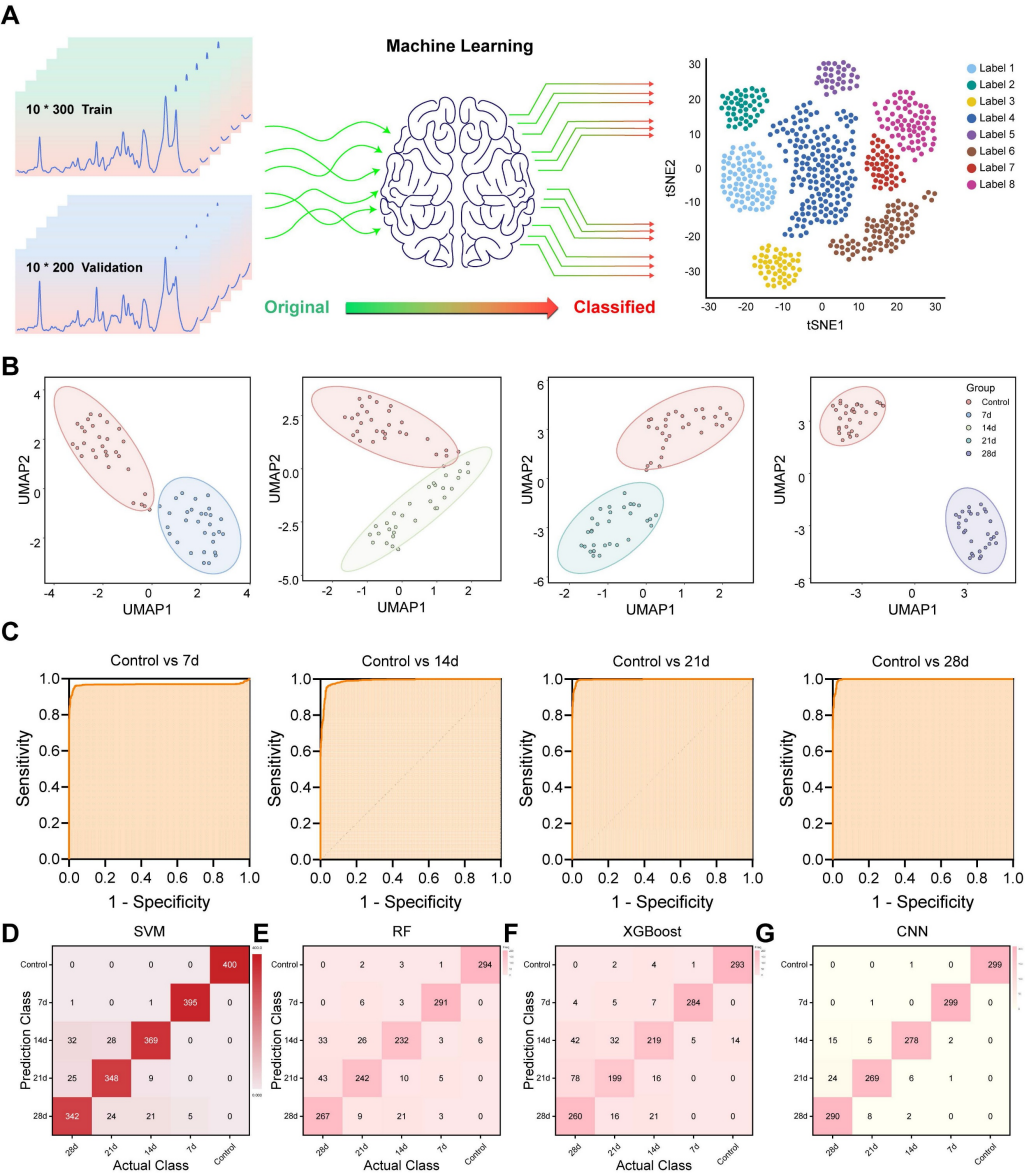
** Flowchart illustrating ML processes applied to Raman spectra of liver tissue.** (**A**) Schematic representation outlining ML methodologies employed. (**B**) UMAP plots for the control and INH-ILI groups. (**C**) ROC plots for the control and INH-ILI groups. (**D**) SVM plots for the control and INH-ILI groups. (**E**) RF test plots for the control and INH-ILI groups. (**F**) XGBoost test plots for the control and INH-ILI groups. (**G**) CNN test plots for the control and INH-ILI groups.

Although spectral differences were visually and statistically evident, manual interpretation remains labor-intensive and subjective. To overcome this limitation and achieve automated tissue classification, multiple ML algorithms were applied to the Raman spectral datasets (Figure 5A). Unsupervised dimensionality reduction techniques, including UMAP and t-SNE, were used to visualize all sample clusters of the control and INH-ILI groups (Figure S6A-B). However, it demonstrated a limited ability to address the nuances among the four separate INH-ILI groups. The INH-ILI groups showed obvious overlap in the UMAP and tSNE, making it very difficult to visually distinguish the clear boundaries between them. Although this overlap may reflect potential biological similarities or the limitations of the selected feature space for a specific visualization task, it does not provide the clear and unique clustering that pairwise comparisons offer to highlight the main contrasts. Therefore, the control group was subjected to UMAP and t-SNE cluster visualization analyses with the INH-ILI 7, 14,21, and 28-day groups respectively (Figure 5B and S7). The spectral data from control and INH-ILI tissues at 7, 14, 21, and 28 days showed clear group separations, with minimal overlap—except between the control and day-14 INH groups, which may reflect mild or reversible injury stages.

For quantitative classification, supervised learning was implemented using a SVM algorithm. The dataset was randomly split into a training set (60%) and a testing set (40%) to evaluate model performance. Unlike UMAP and tSNE, the confusion matrix of SVM (Figure 5D) performs well in classification at different time points. The overall (Figure S6C) and separately (Figure 5C) compared receiver operating characteristic (ROC) curves also demonstrated outstanding diagnostic capabilities, with the area under the curve (AUC) values compared at different time points all exceeding 0.95 (Table S2).

In addition, we randomly divided the dataset into a test set (70%) and a validation set (30%), and conducted comparative analyses of RF, XGBoost, and CNN respectively to further evaluate the model performance. Similar to the SVM results, the classification performance of RF (Figure 5E and S8A), XGBoost (Figure 5F and S8B), and CNN (Figure 5G and S8C) confusion matrices at different time points is all very good. The accuracy rates of the three models are 88.4%, 83.7%, and 95.7% respectively. It is worth mentioning that CNN is significantly superior to RF and XGBoost models, and CNN can be given priority for subsequent studies with larger cohorts. The ROC curve (Figure S8D-F) also demonstrated excellent diagnostic capabilities. The AUC values of different models all exceeded 0.95.

Nonetheless, the integration of Raman spectroscopy with ML significantly enhances diagnostic efficiency, enabling high-throughput and real-time tissue classification without the need for expert interpretation. This automated approach lays the groundwork for future implementation in clinical workflows, particularly in settings where rapid and accurate INH-ILI assessment is critical. Moreover, it demonstrates the potential of Raman-ML systems as decision-support tools in precision hepatology.

### Metabolomic Profiling Reveals Disrupted Pathways in INH-ILI

**Figure 6:**
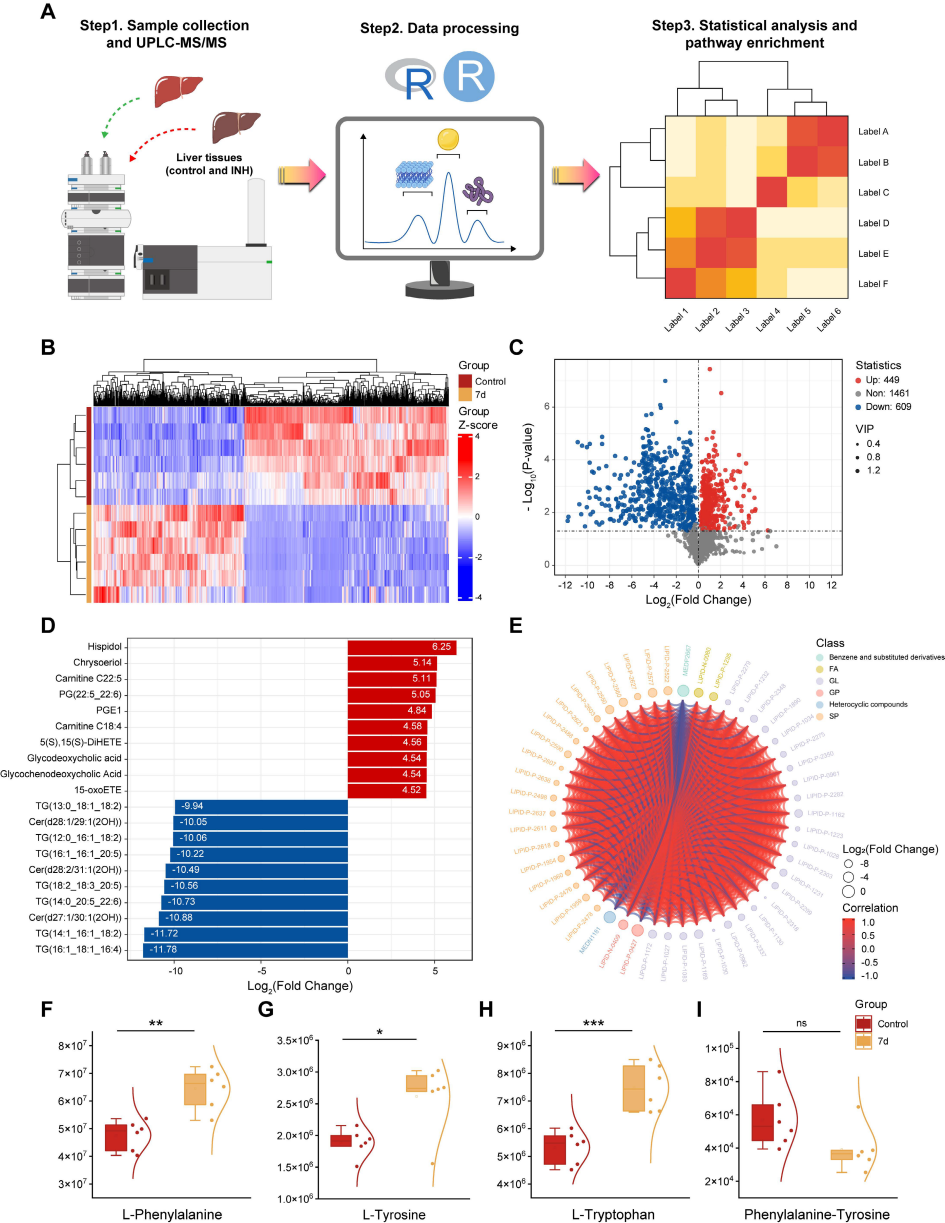
** A widely targeted metabolomics analysis compares the control versus INH-ILI 7-day groups.** (**A**) Schematic diagram of the widely targeted metabolomics analysis process. (**B**) Cluster heatmap illustrating the differential expression of metabolomics between the two groups. (**C**) Volcanic plot depicting differences in metabolites between the two groups. (**D**) The bar chart displays differentially expressed metabolites across both groups. (**E**) Visualization of differentially expressed metabolites through string diagrams. Expression levels of L-phenylalanine (**F**), L-tyrosine (**G**), L-tryptophan (**H**), and Phenylalanine-Tyrosine (I). All the data are presented as mean ± SD. *P < 0.05, **P < 0.01, ***P < 0.001 versus the control group, ns, not significant.

Given the spectral differences identified via Raman analysis, we further explored the molecular mechanisms underlying INH-ILI by performing widely targeted metabolomics on liver tissues from the control group and INH-treated mice at days 7, 14, and 28. UPLC-MS/MS was employed to identify and quantify small-molecule metabolites (Figure 6A). PCA (Figure S9A-C), PLS-DA (Figure S9D-F), and OPLS-DA (Figure S9G-I) demonstrated clear separations between control and INH-ILI groups, confirming distinct metabolic profiles associated with liver injury. Heatmaps (Figure 6B, S10A, and S11A) and volcano plots (Figure 6C, S10B, and S11B) revealed significant dysregulation of numerous metabolites, with 400-700 compounds found to be differentially expressed across time points.

A comprehensive qualitative and quantitative analysis of the identified metabolites was conducted to identify the top 10 upregulated and downregulated metabolites in each group (Figure 6D, S10C, and S11C). Chord diagrams were used to visualize the 50 metabolites with the highest VIP scores (Figure 6E, S10D, and S11D). Details of the top 20 upregulated and downregulated differentially expressed metabolites for each group are provided in the supplementary materials (Figure S12, S13, and S14). Comprehensive findings from differential metabolite screening for each group are summarized in Table S3, S4, and S5.

Among the top perturbed metabolites were key lipids (phosphatidylcholine, triglycerides, oxidized lipids), aromatic amino acids (L-phenylalanine, L-tyrosine, L-tryptophan), and intermediates involved in redox homeostasis. In the initial phase of liver injury (INH-ILI 7-day), the expression levels of L-Phenylalanine (Figure 6F), L-Tyrosine (Figure 6G), and L-Tryptophan (Figure 6H) were increased (*P* < 0.05). In contrast, no significant difference was observed in the Phenylalanine-Tyrosine ratio (Figure 6I) (*P* > 0.05). As the duration of liver injury increased (INH-ILI 14- and 28-day), the expression levels of Phenylalanine-Tyrosine also increased significantly (Figure S15D, H) (*P* < 0.05). At the same time, no notable differences were detected between L-Phenylalanine, L-Tyrosine, and L-Tryptophan levels (Figure S15A-C and Figure S15E-G) (*P* > 0.05). Phenylalanine, tyrosine, and tryptophan are aromatic amino acids essential for human health. In cases of acute liver injury, this phenomenon may be attributed to inhibition of the conversion of phenylalanine to tyrosine 30, 31. Obstruction of the conversion of phenylalanine to tyrosine, such as through the inhibition of phenylalanine hydroxylase activity, results in the accumulation of toxic intermediate metabolites, including phenylpyruvate. This imposes an increased metabolic burden on the liver, diminishes detoxification efficiency, and subsequently accelerates hepatocyte injury and apoptosis via mechanisms such as oxidative stress and mitochondrial dysfunction. Ultimately, these processes contribute to the onset and progression of INH-ILI.

Targeted metabolomics (UPLC-MS/MS) revealed significant disruptions in hepatic lipid metabolism and aromatic amino acid pathways. These metabolic shifts correlate with the Raman spectral changes. Reduced intensity at 1266 cm^-1^ (amide III/lipids) and 1746 cm^-1^ (C=O ester lipids) aligns with disrupted lipid homeostasis. Increased 1203 cm^-1^ (tryptophan/phenylalanine) and 1638 cm^-1^ (H₂O bending) signals reflect amino acid accumulation and cellular edema.

KEGG pathway enrichment analysis (Figure S16) further confirmed that the most significantly affected metabolic pathways in INH-ILI were lipid metabolism, amino acid biosynthesis, and oxidative phosphorylation. These data not only validate the Raman spectroscopic findings but also provide molecular insights into the systemic reprogramming of hepatic metabolism in response to INH-induced stress. By combining spatially resolved spectral imaging with high-throughput metabolomics, we constructed a multidimensional metabolic landscape of INH-ILI, capturing both biochemical identity and distribution. This synergistic approach enhances the discovery of disease-relevant biomarkers and supports mechanistic interpretations of spectral shifts observed in Raman imaging.

## Conclusion

Although this integrated Raman-ML-metabolomics platform demonstrates promising potential in the diagnosis of INH-ILI, its clinical translation still faces several key challenges that must be addressed. These challenges include (1) performing validation studies in human tissues to address the pathophysiological differences between mice and humans; (2) developing interpretable and robust ML models trained on diverse and representative clinical datasets; (3) improving the spatial correlation between metabolic changes and specific histopathological features to enhance tissue resolution; (4) conducting comprehensive time-course metabolomics analyses to align metabolic profiles with Raman spectral changes during disease progression; and (5) optimizing the clinical workflow by integrating solutions compatible with permanently fixed tissue sections and portable detection devices. Addressing these issues is essential for the successful implementation of this platform in real-world clinical settings.

In summary, we established a novel, non-invasive diagnostic platform for INH-ILI by integrating confocal Raman spectroscopy imaging, ML, and metabolomic profiling. Raman spectroscopy enabled the label-free detection of biochemical changes in liver tissue, with ML algorithms achieving high-accuracy classification of injury stages (AUC > 0.95). Furthermore, targeted metabolomics validated the spectroscopic findings and revealed metabolic disruptions involving lipids and aromatic amino acids. This integrative framework not only provides a spatially resolved and real-time approach for identifying hepatotoxicity but also uncovers underlying metabolic mechanisms of INH-ILI. The material-free nature of Raman imaging combined with ML-based spectral interpretation makes this method highly suitable for clinical translation, especially in resource-limited settings where traditional diagnostics are inaccessible or delayed. Our findings lay a foundation for future applications of Raman-based AI diagnostics in drug-induced liver injury and highlight the value of combining imaging and omics technologies to unravel complex disease processes.

## Supplementary Material

Supplementary methods, figures and tables.

## Abbreviations

ANOVA: analysis of variance
AUC: area under the curve
ALT: alanine aminotransferase
AST: aspartate aminotransferase
BW: body weight
CE-MS: capillary electrophoresis-mass spectrometry
CNN: convolutional neural network
3D: three-dimensional
ESI: electrospray ionization
GS-MS: gas chromatography-mass spectrometry
HE: hematoxylin-eosin
INH: isoniazid
INH-ILI: isoniazid-induced liver injury
KEGG: Kyoto Encyclopedia of Genes and Genomes
LC-MS: liquid chromatography-mass spectrometry
LW: liver weight
MS: mass spectrometry
MS/MS: tandem mass spectrometry
NA: numerical aperture
NMR: nuclear magnetic resonance
OCT: optimal cutting temperature
OPLS-DA: orthogonal partial least discriminant analysis
OSC: orthogonal signal correction
PCA: principal component analysis
PLS-DA: partial least squares-discriminant analysis
RF: random forest
ROC: receiver operating characteristic
SD: standard deviation
SPF: standard pathogen-free
SVM: support vector machine
TB: tuberculosis
tSNE: t-distributed stochastic neighbor embedding
UMAP: uniform manifold approximation and projection
UPLC: ultra-performance liquid chromatography
UPLC-MS/MS: ultra-performance liquid chromatography-tandem mass spectrometry
WD: working distance
XGBoost: extreme gradient boosting
