# Supplementary material for: Machine learning-enhanced confocal Raman imaging enables label-free diagnosis and spatial metabolic profiling of isoniazid-induced hepatotoxicity
Source: Theranostics. 2025 Aug 30;15(18):9663–77. doi: 10.7150/thno.119785 (PMC12486251; doi:10.7150/thno.119785)
Supplement: Supplementary file 1 — Supplementary methods, figures and tables. [file thnov15p9663s1.pdf]

Supplementary material

**Machine learning-enhanced confocal Raman imaging enables label-free diagnosis and spatial metabolic profiling of isoniazid-induced hepatotoxicity**

*Shimei Wang[b], Xiaoren Wang[c], Xudong Cui[d], Xiaotong Xie[b], Zhu Zhu[b], Tomii Ayaka[b], Renxing Song[e], Liping Zhou[e], Jin Sun[e], Li Zhang[e], Ruisheng Ge[g], Lei Yu[b,*], Yang Li[a,e,f,*]*

[a] Research Center for Innovative Technology of Pharmaceutical Analysis, College of Pharmacy, Harbin Medical University, Harbin 150081, PR China

[b] Department of Infectious Disease, The Fourth Affiliated Hospital of Harbin Medical University, Harbin 150001, PR China

[c] Department of Infectious Disease, The Second Affiliated Hospital of Harbin Medical University, Harbin 150001, PR China

[d] Cardiovascular Center, Inner Mongolia Peoples's Hospital, Hohhot 010010, PR China

[e] State Key Laboratory of Frigid Zone Cardiovascular Diseases (SKLFZCD), College of Pharmacy, Harbin Medical University, Harbin 150081, PR China

[f] Research Unit of Health Sciences and Technology (HST), Faculty of Medicine University of Oulu, Oulu 90220, Finland

[g] Department of Pathology, The Fourth Affiliated Hospital of Harbin Medical University, Harbin 150001, PR China

[*] **Corresponding authors**

Yang Li, Research Center for Innovative Technology of Pharmaceutical Analysis, College of Pharmacy, Harbin Medical University, Harbin 150081, PR China, Tel.: +86-173-4567-8922, E-mail: liy@hrbmu.edu.cn.

Lei Yu, Department of Infectious Disease, The Fourth Affiliated Hospital of Harbin Medical University, Harbin 150001, PR China, Tel.: +86-138-0453-5457, E-mail: YL@hrbmu.edu.cn.

## Animal model

The standard conversion formula is: HED (mg/kg) = Animal Dose (mg/kg) × (Animal Km / Human Km) [1]. For mice to human conversion: Mouse Km factor = 3, Human Km factor = 37. The standard daily dose of isoniazid for adults is usually 5mg/kg [2]. Therefore: Animal Dose = 5 mg/kg × (37/3) ≈ 61.7 mg/kg. This calculated Animal Dose (~61.7 mg/kg) falls well within the standard therapeutic range for INH in humans (5 mg/kg/day) and is notably below the doses commonly associated with clinical hepatotoxicity risk (often >10 mg/kg/day, especially in slow acetylators or with concomitant risk factors like rifampicin co-administration or pre-existing liver disease). While the absolute mouse dose (100 mg/kg) appears high compared to the human therapeutic dose (5 mg/kg), the HED calculation confirms that the *relative systemic exposure* in our mouse model (~ 61.7 mg/kg HED) is clinically relevant and approximates doses known to cause hepatotoxicity in susceptible human populations.

In addition, in rodent studies, especially in rat studies, the commonly used dose of INH obtained through oral exposure is 50-55 mg/kg, with varying durations [3, 4]. We now supplement the fundamental principles by calculating the HED based on body surface area normalized in accordance with FDA guidelines and widely accepted principles [1]. The standard conversion formula is: Animal rat (mg/kg) = Animal mouse (mg/kg) × (Animal mouse Km/Animal rat Km). For rat to mouse conversion: Rat Km factor = 6, Mouse Km factor = 3. Therefore: Animal mouse = 50-55 mg/kg / (3/6) = 100-110 mg/kg, that is, the dose administered in mice is twice that given in rats. This provides a strong pharmacokinetic/pharmacodynamic basis for our selected dose in reproducing INH-ILI.

## UPLC-MS/MS Metabolomics Experimental Procedures and Data Analysis

### Methods for extraction of hydrophilic compounds

Liver tissue samples (20 mg ± 1 mg) were individually weighed into labeled centrifuge

tubes. Each sample was homogenized with a steel ball using a ball mill (30 Hz, 20 s). After centrifugation (3000 rpm, 30 s, 4°C), 400 μL of 70% methanol-water solution (containing an internal standard) was added, followed by shaking (2500 rpm, 5 min) and incubation on ice (15 min). Subsequent centrifugation (12000 rpm, 10 min, 4°C) yielded a supernatant; 300 μL of this supernatant was transferred to a new tube and stored at -20°C for 30 min. A final centrifugation step (12000 rpm, 3 min, 4°C) provided a supernatant from which 200 μL was transferred to sample vials for analysis.

**UPLC Conditions of hydrophilic compounds**

Chromatographic separation was achieved using a Waters ACQUITY UPLC HSS T3 C18 column (1.8 μm, 2.1 mm × 100 mm). The mobile phases were as follows: Phase A, ultra-pure water (0.1% formic acid); Phase B, acetonitrile (0.1% formic acid). The elution gradient was as follows: 0 min, 95:5 (water: acetonitrile, V/V); 2.0 min, 80:20 (V/V); 5.0 min, 40:60 (V/V); 6.0 min, 1:99 (V/V); 7.5 min, 1:99 (V/V); 7.6 min, 95:5 (V/V); 10.0 min, 95:5 (V/V). The flow rate was 0.4 mL/min, the column temperature was 40°C, and the injection volume was two μL.

**ESI-Q TRAP-MS/MS of hydrophilic compounds**

Electrospray ionization (ESI) was performed at 500 °C, with a mass spectrum voltage of 5500 V (positive mode) or -4500 V (negative mode). Ion source gases were set to 55 psi (Gas I) and 60 psi (Gas II), and the curtain gas (CUR) to 25 psi. Collision-activated dissociation (CAD) was set to high. Optimized declustering potential (DP) and collision energy (CE) parameters were used for each ion pair in the triple quadrupole-linear ion trap (QTRAP) mass spectrometer.

**Methods for extraction of hydrophobic compounds**

Lipid extraction involved adding 1 mL of internal standard-containing methyl tert-butyl ether: methanol (3:1, V/V) to the sample, followed by swirling (15 min). After adding 200 μL of water and swirling (1 min), centrifugation (12000 rpm, 10 min, 4°C) was performed. 200 μL of the supernatant was transferred, dried completely, and then

reconstituted in 200 μL of acetonitrile: isopropyl alcohol (1:1, V/V) by swirling (3 min). A final centrifugation (12000 rpm, 3 min, 4°C) prepared the supernatant for UPLC-MS/MS analysis.

**UPLC Conditions of hydrophobic compounds**

A Thermo Accucore™ C30 column (2.6 μm, 2.1 mm × 100 mm) was used for chromatographic separation. Mobile phase: Phase A, acetonitrile/water (60/40, V/V) (0.1% formic acid, 10 mmol/L ammonium formate); Phase B, acetonitrile/isopropyl alcohol (10/90, V/V) (0.1% formic acid, 10 mmol/L ammonium formate). Elution gradient: 0 min, 80:20 (A/B, V/V); 2 min, 70:30 (V/V); 4 min, 40:60 (V/V); 9 min, 15:85 (V/V); 14 min, 10:90 (V/V); 15.5 min, 5:95 (V/V); 17.3 min, 5:95 (V/V); 17.5 min, 80:20 (V/V); 20 min, 80:20 (V/V). Flow rate: 0.35 mL/min; Column temperature: 45°C; Injection volume: 2 μL.

**ESI-Q TRAP-MS/MS of hydrophobic compounds**

ESI was performed at 500°C, using a mass spectrum voltage of 5500 V (positive ion mode) and -4500 V (negative ion mode). Ion source gases were set to 45 psi (Gas 1) and 55 psi (Gas 2), with the CUR was set to 35 psi. The CAD parameter was set to medium. Each ion pair was scanned in the QTRAP system using optimized DP and CE values.

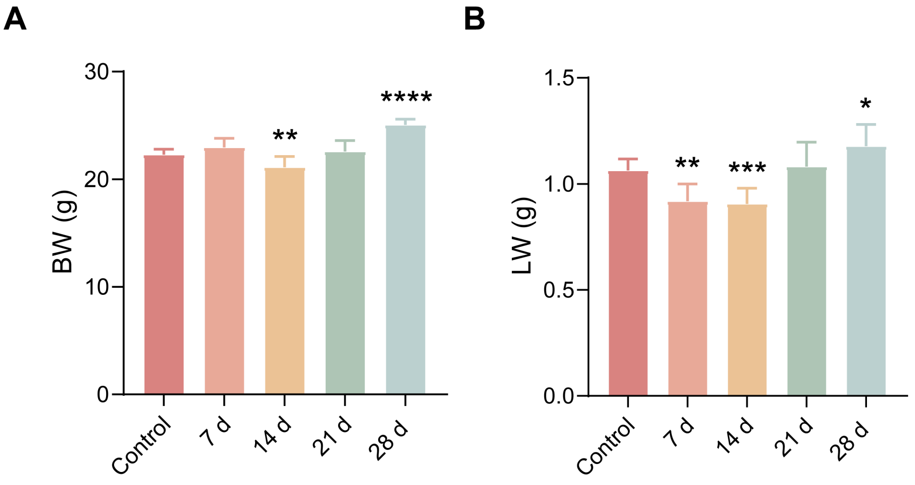


**Figure S1. Comparison of BW and LW between the control and INH-ILI groups.** (**A**) BW and (**B**) LW measurements for the control and INH-ILI groups, respectively (n = 10). All the Data are presented as mean ± SD. *$P < 0.05$, **$P < 0.01$, ***$P < 0.001$, ****$P < 0.0001$ versus the control group.

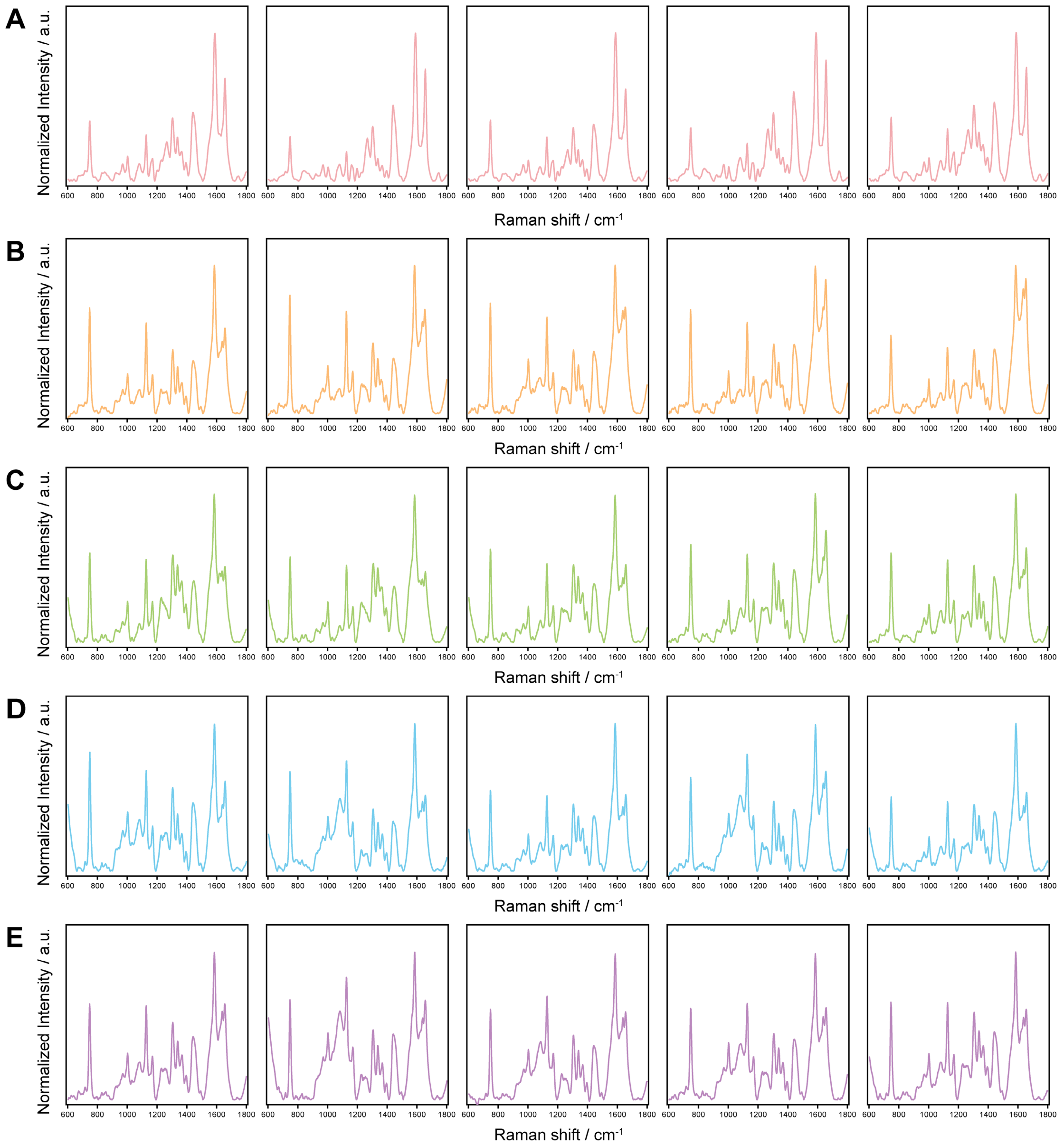


**Figure S2. Representative Raman spectra are obtained from liver tissue samples in control and INH-ILI groups.** The data for the control group are shown in (**A**), followed by the INH-ILI 7- (**B**), 14- (**C**), 21- (**D**), and 28-day (**E**) groups, respectively.

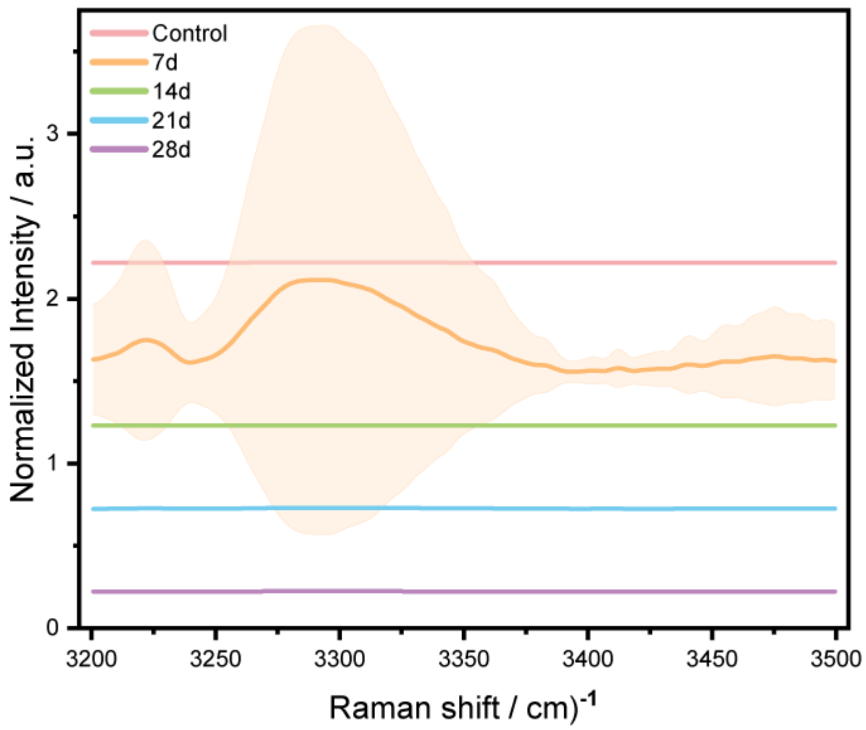


**Figure S3. The average Raman spectrum within the range of 3200-3500 cm⁻¹.**

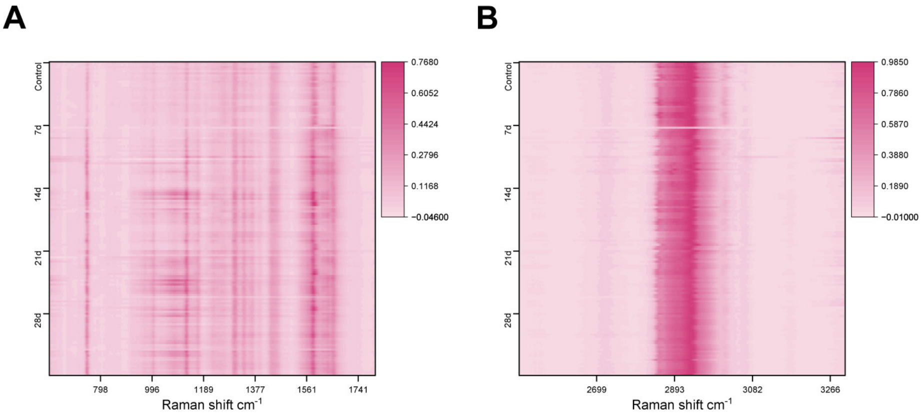


**Figure S4. Heat maps depicting Raman spectral intensities for both control and INH-ILI groups.** (**A**) Heat map in the range of 600-1800 cm$^{-1}$. (**B**) Heat map in the range of 2500-3300 cm$^{-1}$.

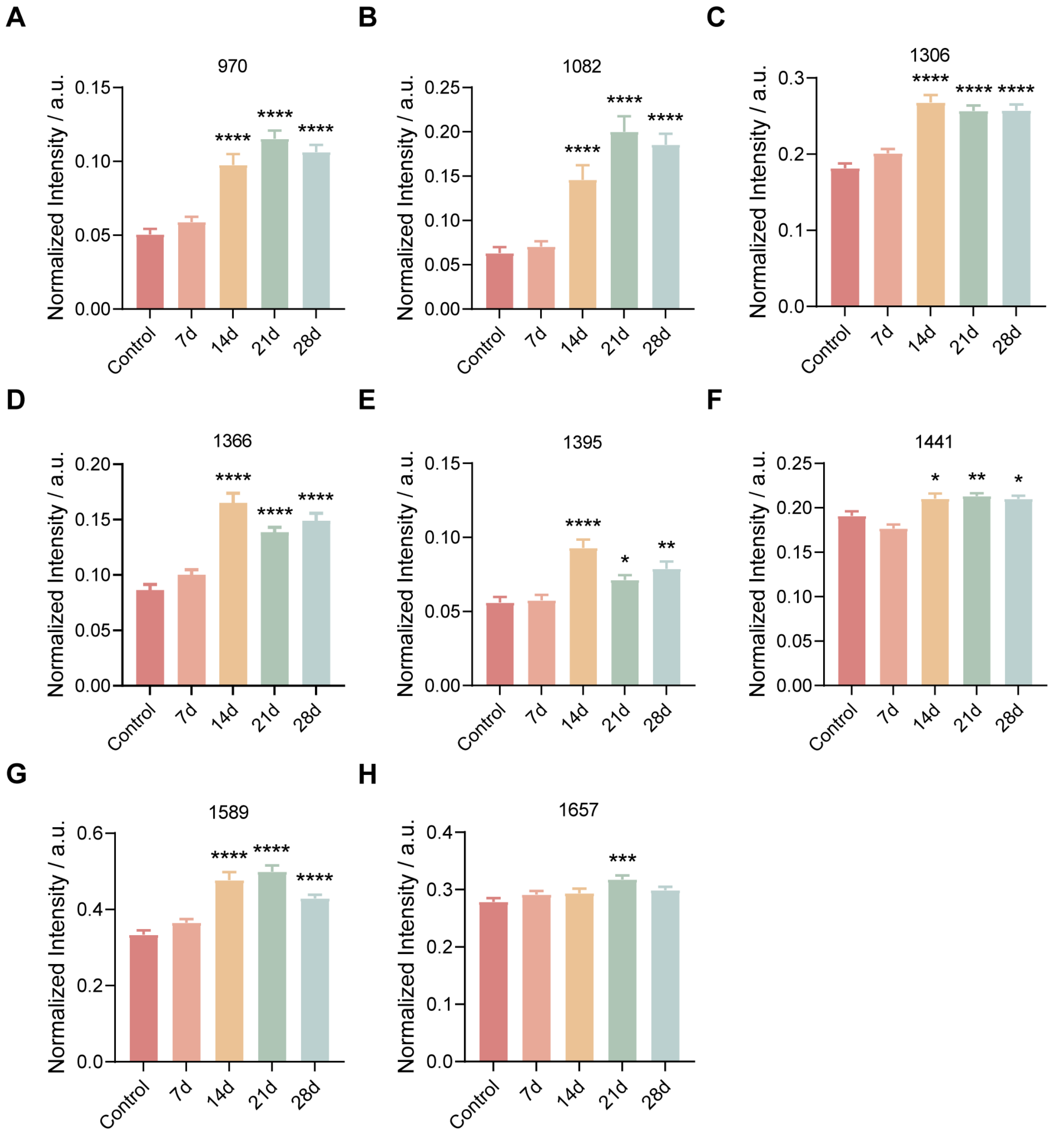


**Figure S5. Partial Raman spectral difference in the characteristic peak intensities between the control and INH-ILI groups.** Peak intensities at (**A**) 970, (**B**) 1082, (**C**) 1306, (**D**) 1366, (**E**) 1395, (**F**) 1441, (**G**) 1589, and (**H**) 1657 $cm^{-1}$ for both control and INH-ILI groups, respectively. All the Data are presented as mean ± SD. **P* < 0.05, ***P* < 0.01, ****P* < 0.001, *****P* < 0.0001 versus the control group.

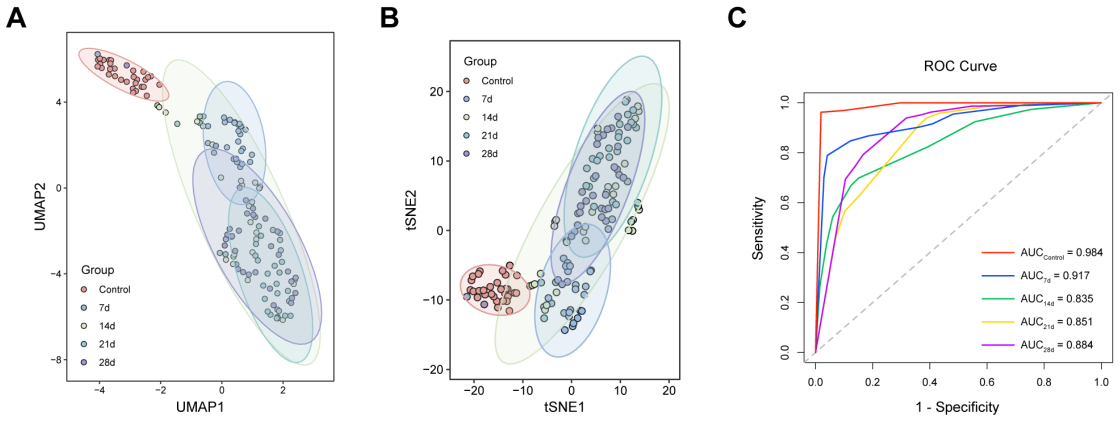


**Figure S6. UAMP, tSNE, and ROC plots for the control and INH-ILI groups. (A)** UMAP plots for the control and INH-ILI groups. **(B)** tSNE plots for the control and INH-ILI groups. **(C)** ROC plots for the control and INH-ILI groups.

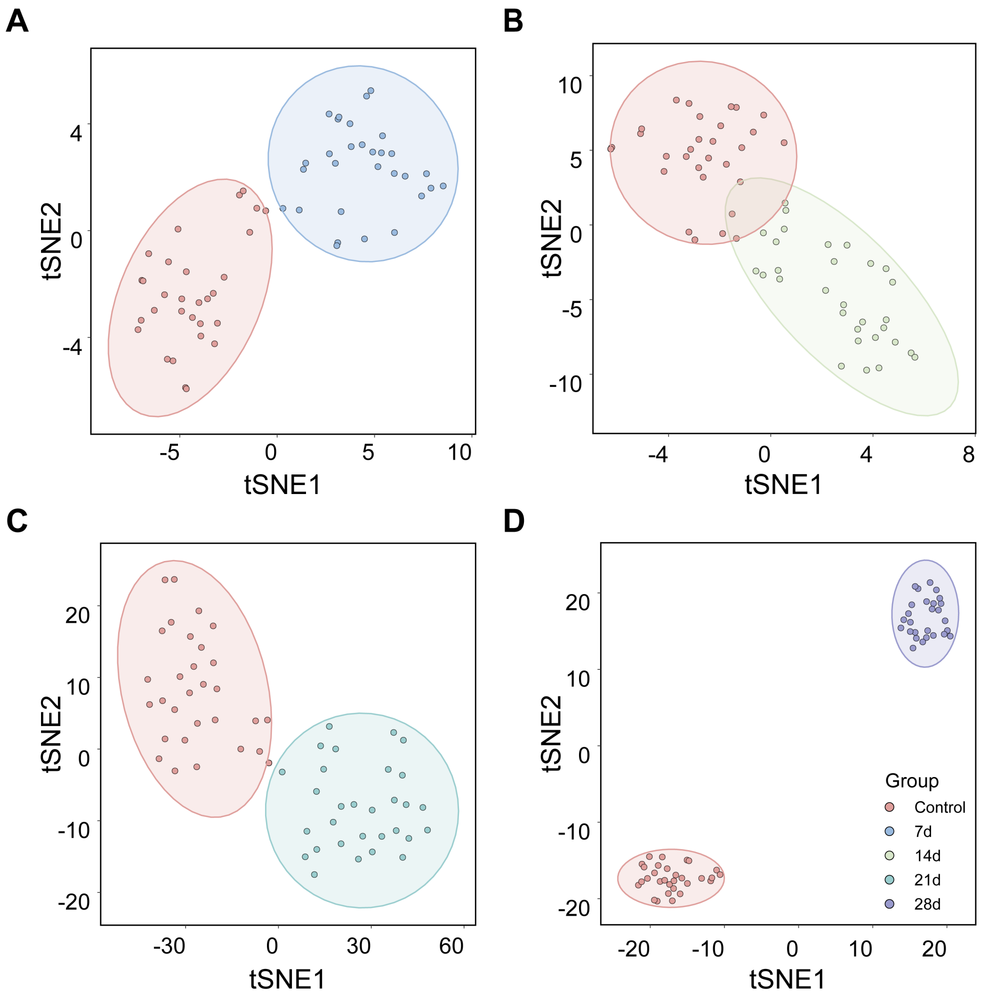


**Figure S7. t-SNE plots for the control and INH-ILI groups.** Comparisons include control versus INH-ILI 7- (**A**), 14- (**B**), 21- (**C**), and 28-day (**D**) groups, respectively.

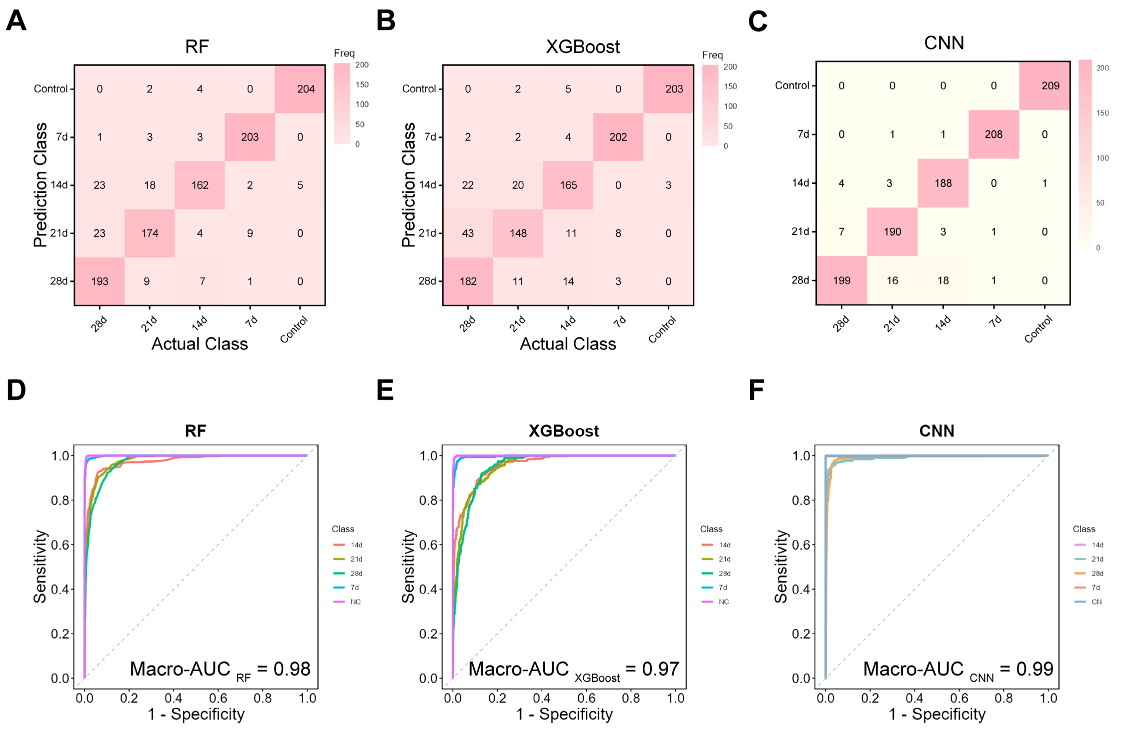


**Figure S8. RF, XGBoost, and CNN verification plots and ROC curves of the control and INH-ILI groups.** **(A)** RF verification plots for the control and INH-ILI groups. **(B)** XGBoost verification plots for the control and INH-ILI groups. **(C)** CNN verification plots for the control and INH-ILI groups. **(D)** RF ROC curves for the control and INH-ILI groups. **(E)** XGBoost ROC curvesROC curves for the control and INH-ILI groups. **(F)** CNN ROC curves for the control and INH-ILI groups.

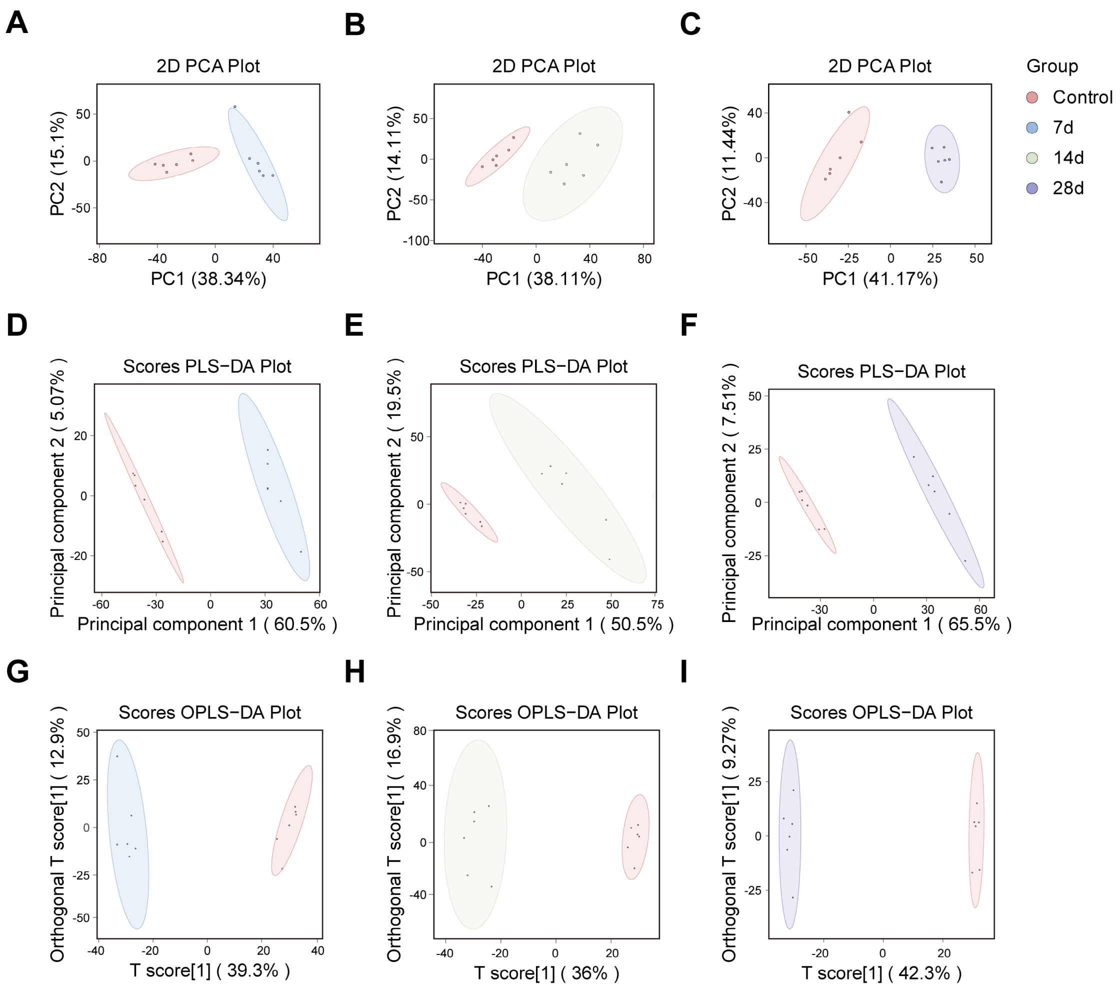


**Figure S9. PCA, PLS-DA, and OPLS-DA plots for the control and INH-ILI groups.** (**A-C**) PCA plots for the control group versus the INH-ILI 7-, 14-, and 28-day groups, respectively. (**D-F**) PLS-DA plots for the control group versus the INH-ILI 7-, 14-, and 28-day groups, respectively. (**G-I**) OPLS-DA plots for the control group versus the INH-ILI 7-, 14-, and 28-day groups, respectively.

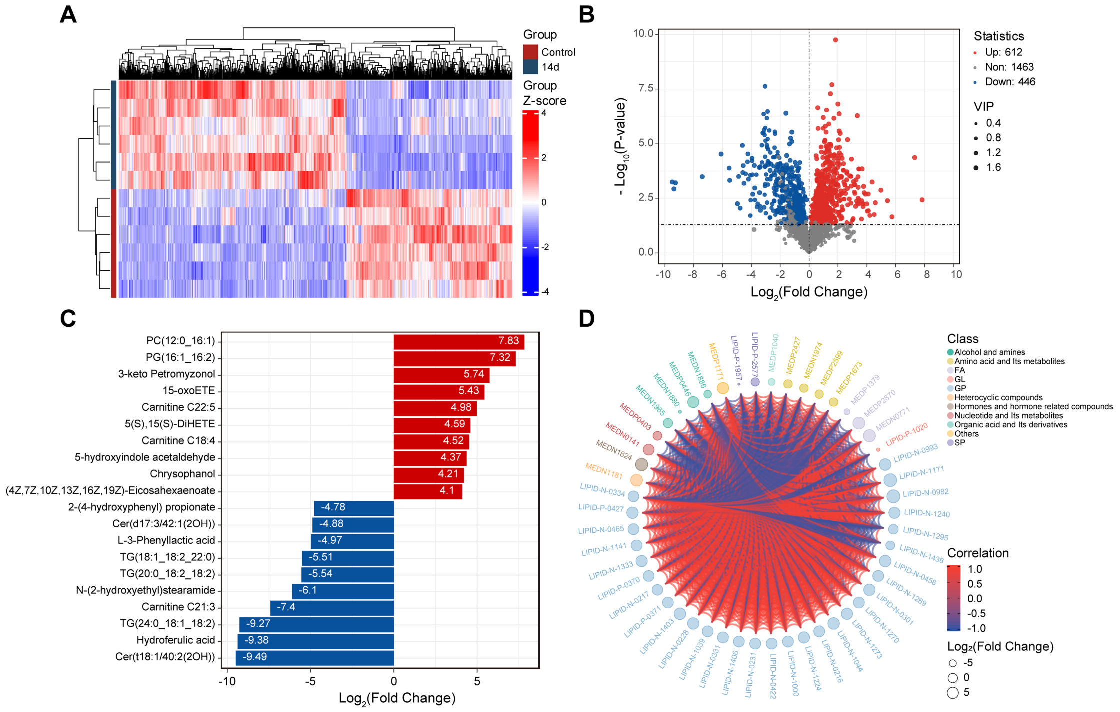


**Figure S10. A widely targeted metabolomics analysis compares the control versus INH-ILI 14-day groups.** (**A**) Cluster of heatmaps of differential metabolites of the control versus the INH-ILI 14-day groups. Red and blue stand for high and low metabolomic expression levels, respectively. (**B**) Volcano plots of differential metabolites of the control versus the INH-ILI 14-day groups. Each point in the volcanic map represents a metabolite, where red and blue respectively represent upregulated and downregulated differential metabolites, and gray represents the metabolites that are detected but not significantly different; the abscissa represents the logarithm of the multiple of the relative content difference of a metabolite between the two groups of samples ($log_2$(Fold Change)). The larger the absolute value of the abscissa, the more significant the relative difference in the content of the substance between the two groups of samples. The ordinate represents the significance level of the difference, and the point size represents the VIP value. (**C**) The bar chart of differential metabolites of the control versus the INH-ILI 14-day groups. The differential metabolites' $log_2$(Fold Change) is plotted on the abscissa. Red and blue represent the upregulation and downregulation of metabolite levels, respectively. (**D**) The string diagrams of differential metabolites of the control versus the INH-ILI 14-day groups. The outermost layer represents the names of differentially expressed metabolites, the size of the points represents the $log_2$(Fold Change) value of the corresponding differentially expressed metabolite, different colors represent different categories of corresponding differentially expressed metabolites, and the thickness of the lines represents the magnitude of the Pearson correlation coefficient between corresponding differentially expressed metabolites, with red lines representing positive correlation and blue lines representing negative correlation.

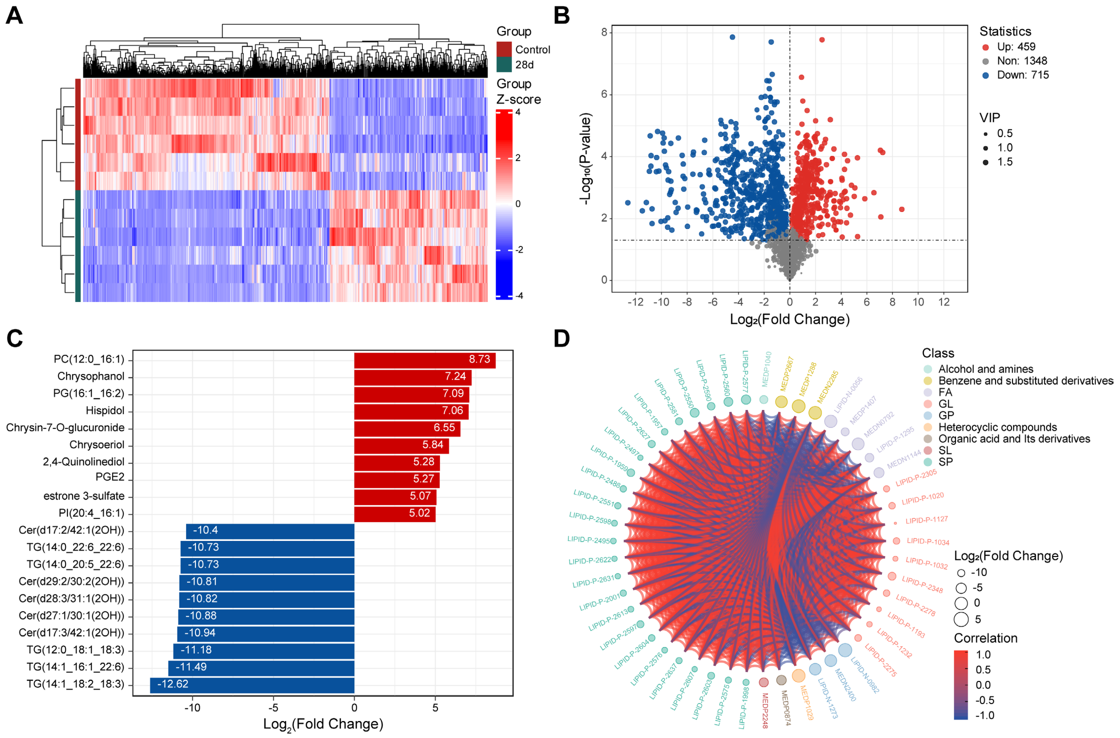


**Figure S11. A widely targeted metabolomics analysis compares the control versus INH-ILI 28-day groups.** (**A**) Cluster of heatmaps of differential metabolites of the control versus the INH-ILI 28-day groups. Red and blue stand for high and low metabolomic expression levels, respectively. (**B**) Volcano plots of differential metabolites of the control versus the INH-ILI 28-day groups. Each point in the volcanic map represents a metabolite, where red and blue respectively represent upregulated and downregulated differential metabolites, and gray represents the metabolites that are detected but not significantly different; the abscissa represents the logarithm of the multiple of the relative content difference of a metabolite between the two groups of samples ($log_2$(Fold Change)). The larger the absolute value of the abscissa, the more significant the relative difference in the content of the substance between the two groups of samples. The ordinate represents the significance level of the difference, and the point size represents the VIP value. (**C**) The bar chart of differential metabolites of the control versus the INH-ILI 28-day groups. The differential metabolites' $log_2$(Fold Change) is plotted on the abscissa. Red and blue represent the upregulation and downregulation of metabolite levels, respectively. (**D**) The string diagrams of differential metabolites of the control versus the INH-ILI 28-day groups. The outermost layer represents the names of differentially expressed metabolites, the size of the points represents the $log_2$(Fold Change) value of the corresponding differentially expressed metabolite, different colors represent different categories of corresponding differentially expressed metabolites, and the thickness of the lines represents the magnitude of the Pearson correlation coefficient between corresponding differentially expressed metabolites, with red lines representing positive correlation and blue lines representing negative correlation.

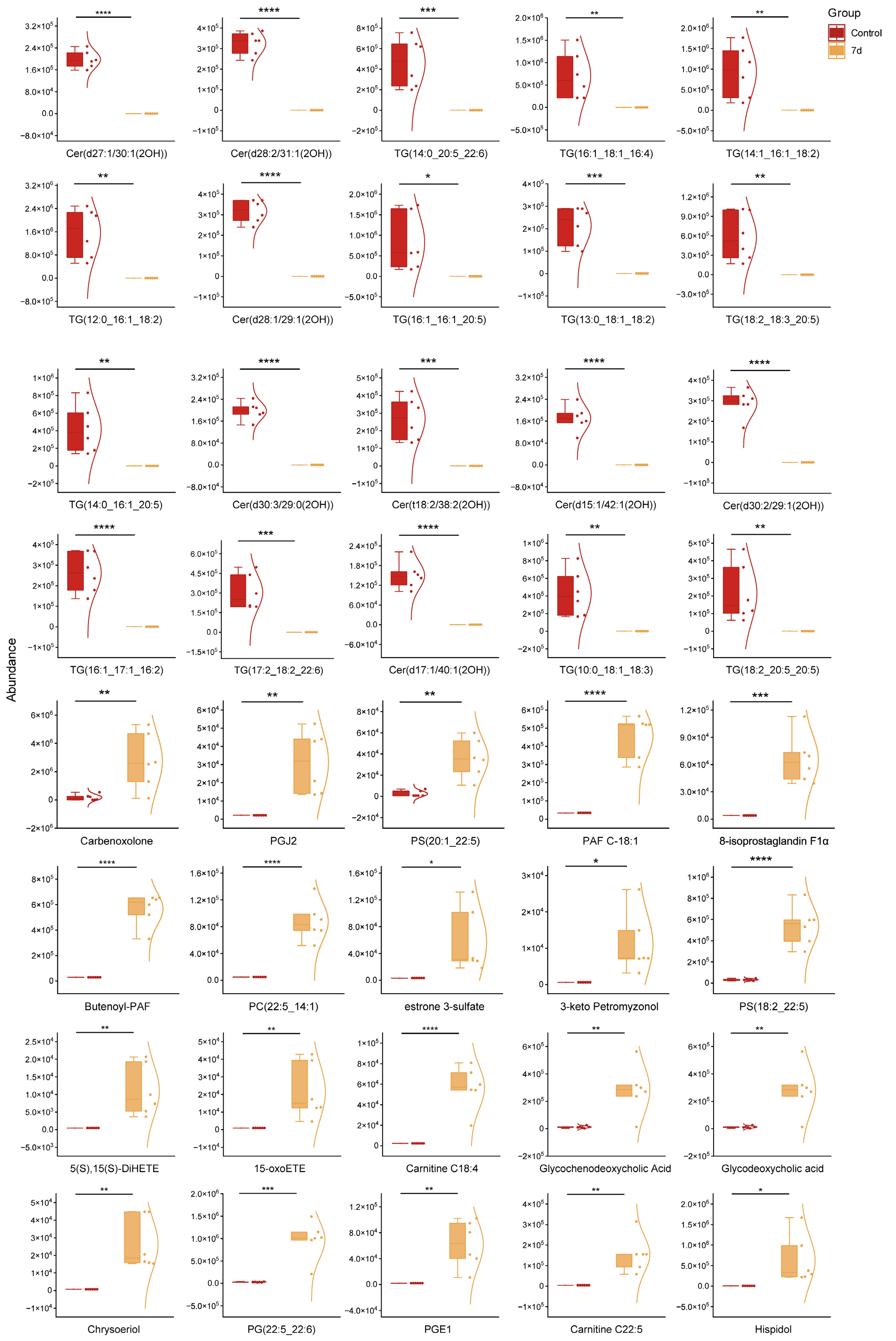


**Figure S12. The boxplots illustrate the representative differential metabolites between the control and INH-ILI 7-day groups.** The abscissa represents the names of differential metabolites, while the ordinate indicates their relative content. All the Data are presented as mean ± SD. *$P < 0.05$, **$P < 0.01$, ***$P < 0.001$, ****$P < 0.0001$ versus the control group.

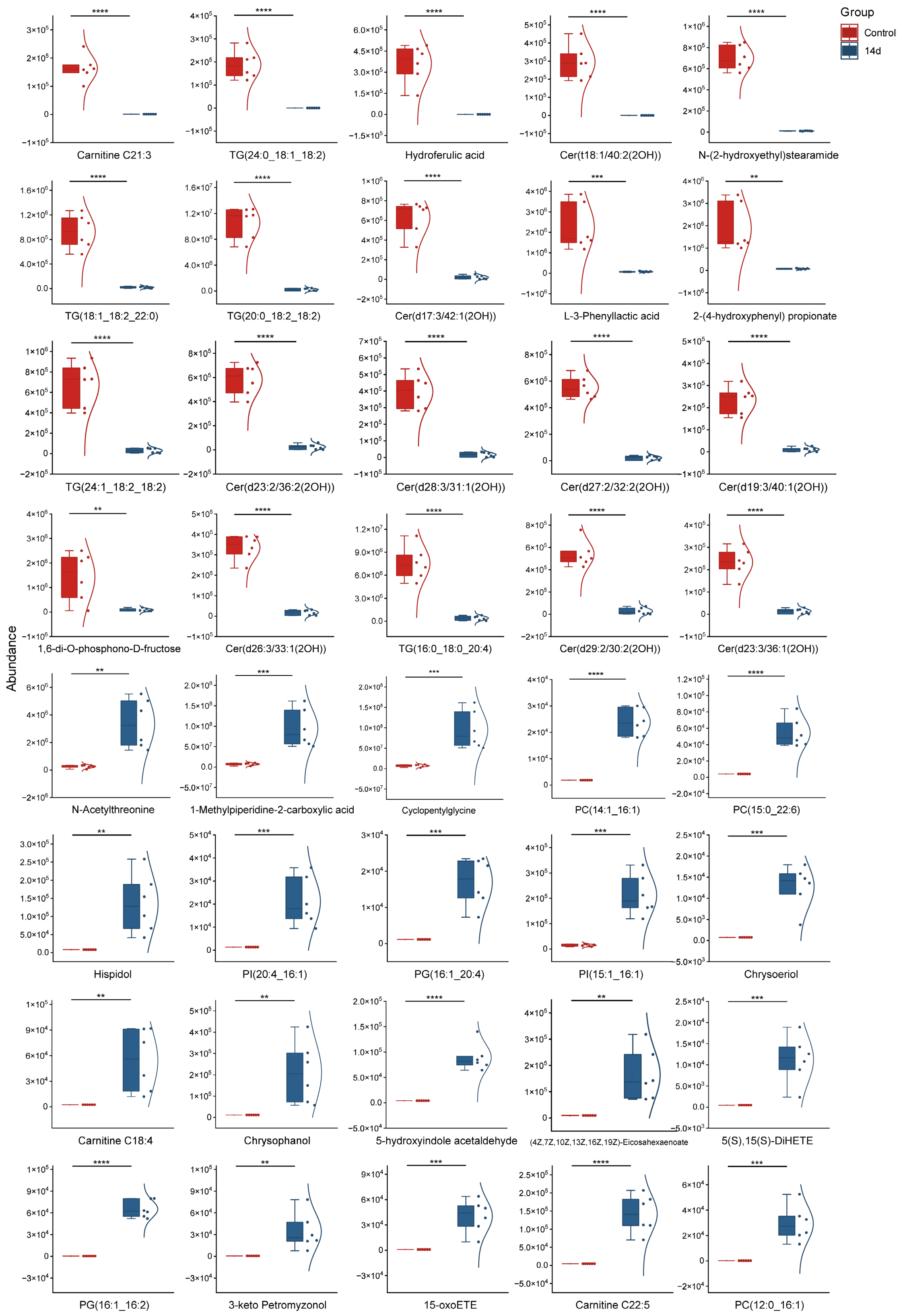


**Figure S13. The boxplots illustrate the representative differential metabolites between the control and INH-ILI 14-day groups.** The abscissa represents the names of differential metabolites, while the ordinate indicates their relative content. All the Data are presented as mean ± SD. **P* < 0.05, ***P* < 0.01, ****P* < 0.001, *****P* < 0.0001 versus the control group.

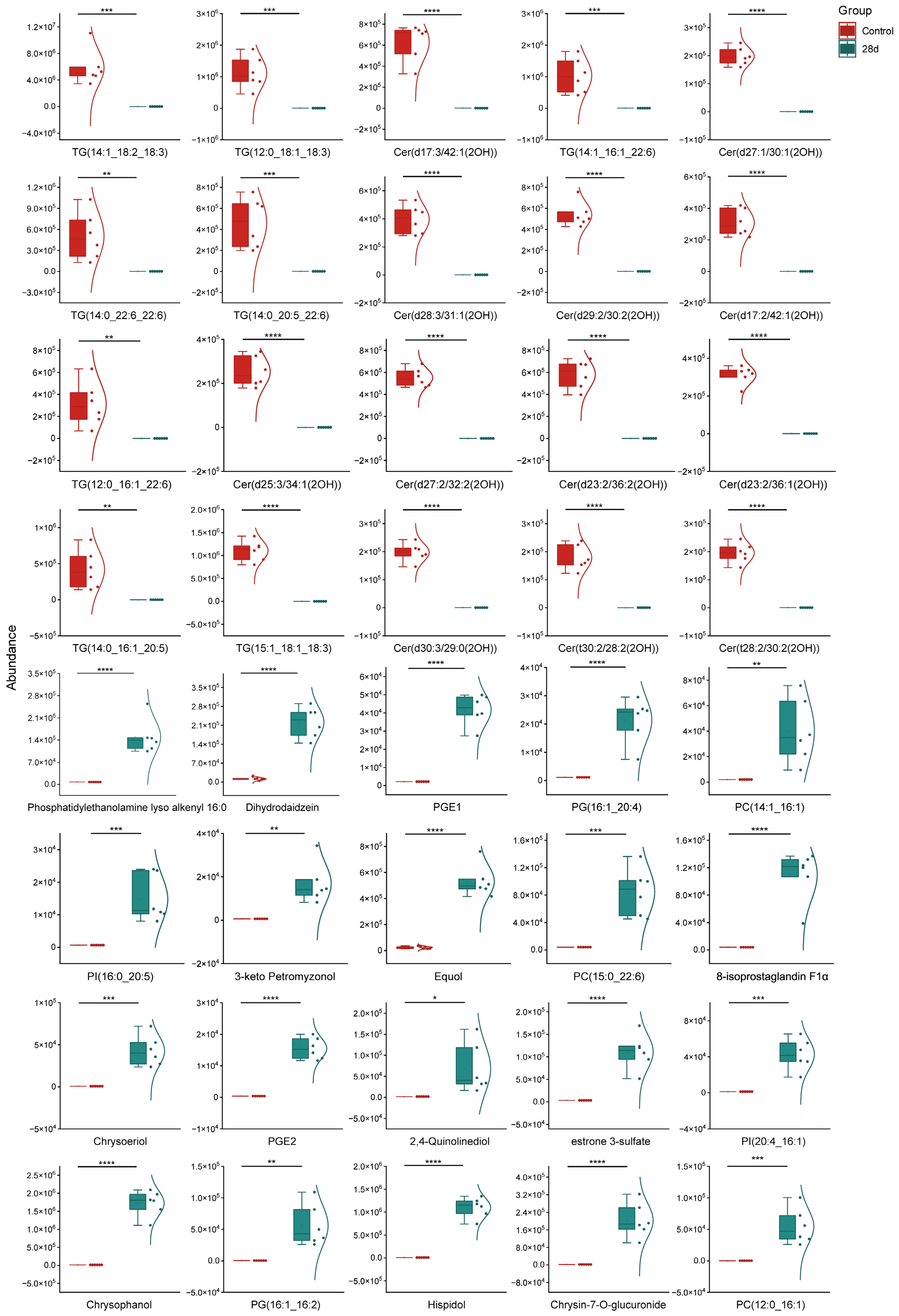


**Figure S14. The boxplots illustrate the representative differential metabolites between the control and INH-ILI 28-day groups.** The abscissa represents the names of differential metabolites, while the ordinate indicates their relative content. All the Data are presented as mean ± SD. $^{*}P < 0.05$, $^{**}P < 0.01$, $^{***}P < 0.001$, $^{****}P < 0.0001$ versus the control group.

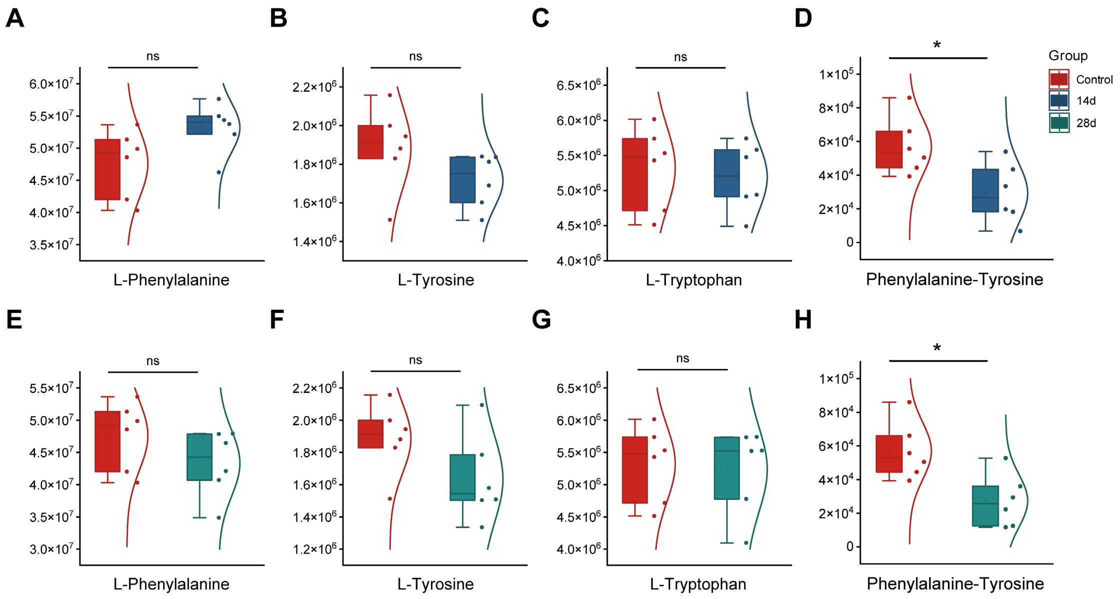


**Figure S15. Expression levels of L-phenylalanine, L-tyrosine, L-tryptophan, and Phenylalanine-Tyrosine were compared between the control versus INH-ILI 14- and 28-day groups.** The abscissa represents the names of differential metabolites, while the ordinate indicates their relative content. All the Data are presented as mean ± SD. **P* < 0.05 versus the control group, ns, not significant.

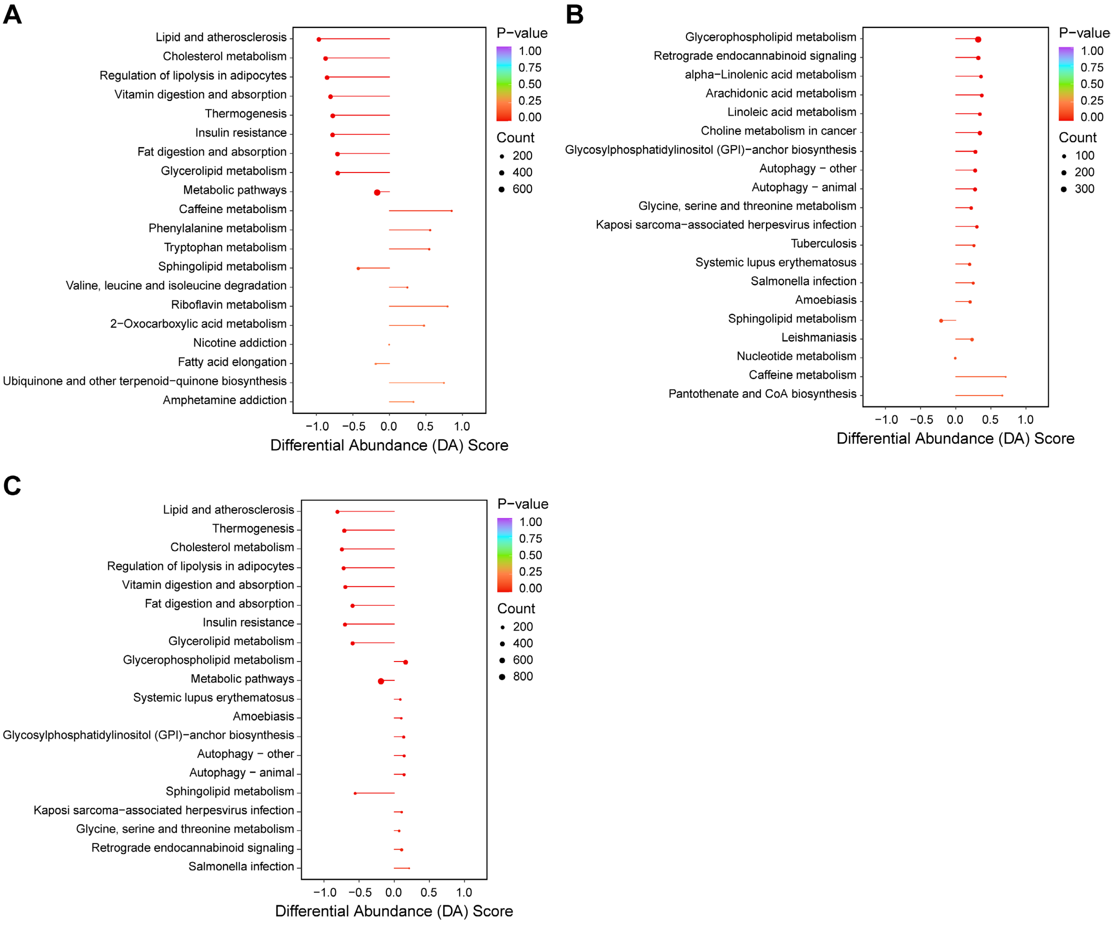


**Figure S16. The KEGG differential abundance scores for the control versus INH-ILI 7-, 14-, and 28-day groups.** KEGG of control versus INH-ILI 7- (**A**), 14- (**B**), and 28-day (**C**) groups. The ordinate denotes different pathway names sorted by *P*-value, while the abscissa reflects differential abundance scores (DA scores). The DA score encapsulates total changes in all metabolites within a given pathway: a score of 1 signifies that all identified metabolites in this pathway exhibit an up-regulated expression trend, whereas a score of -1 indicates a down-regulated trend. The length of each line segment corresponds to the absolute value of the DA score; more significant segments suggest more significant overall down-regulation when positioned to the left of the central axis or up-regulation when on the right side. Additionally, dot size at segment endpoints correlates with metabolite quantity within pathways- the larger dots indicate more metabolites present. Color coding for line segments and dots reflects *P*-value magnitude: red hues signify smaller *P*-values, while purple shades indicate larger ones.

**Table S1.** The leading positions of the Raman vibration peaks and the corresponding representative compounds in control and INH-ILI liver tissues.

| Position | Vibrational mode | Major assignment |
|---|---|---|
| 749 [5-8] | Symmetric ring breathing | Tryptophan |
| 970 [9] | Phosphate monoester groups of phosphorylated | Proteins and nucleic acids |
| 1004 [8, 10] | C-C symmetric ring stretching | Phenylalanine |
| 1082 [11] | $PO^{-}_{2}$ symmetric stretching | Nucleic acids and phospholipids |
| 1128 [6, 12, 13] | C-C stretching | Lipids |
| 1169 [6] | C-C stretching | Tyrosine |
| 1203 [14] | $C\text{-}C_6H_5$ stretching | L-tryptophan and Phenylalanine |
| 1230 [9] | Antisymmetric phosphate stretching | Amide III (β-pleated sheet) |
| 1266 [15] | C=C bending | Lipids |
| 1306 [6, 8] | $CH_3/CH_2$ twisting or bending | Collagen and phospholipids |
| 1338 [16] | $CH_2$ deformation | Collagen |
| 1366 [10] | $CH_3$ stretching | Phospholipids |
| 1395 [5, 17] | CH rocking | Tryptophan |
| 1441 [18] | $CH_3$ scissoring & $CH_2$ bending | Lipids |
| 1589 [5, 6] | C=C bending | Phenylalanine and tryptophan |
| 1638 [7, 10] | | Intermolecular bending mode of water |
| 1657 [9, 19-22] | C=C stretching | Fatty acids, triglycerides |
| 1746 [7] | C=O stretching | Lipids |

**Table S2.** AUC value of ROC curve in control and INH-ILI groups.

| | **7d** | **14d** | **21d** | **28d** |
|---|---|---|---|---|
| **Control** | 0.9677 | 0.9895 | 0.9976 | 0.9974 |

**Table S3.** Results of differential metabolite screening between the control and the INH-ILI 7-day groups.

| **Index** | **Compounds** | **Type** |
|---|---|---|
| MEDP2765 | PAF C-18:1 | Up |
| LIPID-N-1351 | PS (20:1_22:5) | Up |
| MEDN0797 | PGJ2 | Up |
| MEDN2334 | Carbenoxolone | Up |
| MEDN1082 | 8-isoprostaglandin F1α | Up |
| LIPID-N-1360 | PS (18:2_22:5) | Up |
| MEDN1824 | 3-keto Petromyzonol | Up |
| MEDN0536 | estrone 3-sulfate | Up |
| LIPID-N-0301 | PC (22:5_14:1) | Up |
| MEDP1171 | Butenoyl-PAF | Up |
| MEDN0771 | 15-oxoETE | Up |
| MEDN1904*213 | Glycodeoxycholic acid | Up |
| MEDN0113*213 | Glycochenodeoxycholic Acid | Up |
| MEDN0776 | 5(S),15(S)-DiHETE | Up |
| MEDP1393 | Carnitine C18:4 | Up |
| MEDN0792 | PGE1 | Up |
| LIPID-N-1224 | PG (22:5_22:6) | Up |
| MEDP2870 | Carnitine C22:5 | Up |
| MEDP2255 | Chrysoeriol | Up |
| MEDP1029 | Hispidol | Up |
| LIPID-P-2318 | TG (16:1_18:1_16:4) | Down |
| LIPID-P-1030 | TG (14:1_16:1_18:2) | Down |
| LIPID-P-2607 | Cer (d27:1/30:1(2OH)) | Down |
| LIPID-P-1232 | TG (14:0_20:5_22:6) | Down |
| LIPID-P-1223 | TG (18:2_18:3_20:5) | Down |
| LIPID-P-2621 | Cer (d28:2/31:1(2OH)) | Down |

| Index | Compounds | Type |
|---|---|---|
| LIPID-P-1166 | TG (16:1_16:1_20:5) | Down |
| LIPID-P-0961 | TG (12:0_16:1_18:2) | Down |
| LIPID-P-2618 | Cer (d28:1/29:1(2OH)) | Down |
| LIPID-P-2279 | TG (13:0_18:1_18:2) | Down |
| LIPID-P-1960 | Cer (t18:2/38:2(2OH)) | Down |
| LIPID-P-2636 | Cer (d30:2/29:1(2OH)) | Down |
| LIPID-P-2637 | Cer (d30:3/29:0(2OH)) | Down |
| LIPID-P-1130 | TG (14:0_16:1_20:5) | Down |
| LIPID-P-2476 | Cer (d15:1/42:1(2OH)) | Down |
| LIPID-P-1028 | TG (10:0_18:1_18:3) | Down |
| LIPID-P-2488 | Cer (d17:1/40:1(2OH)) | Down |
| LIPID-P-2350 | TG (17:2_18:2_22:6) | Down |
| LIPID-P-1034 | TG (16:1_17:1_16:2) | Down |
| LIPID-P-1238 | TG (18:2_20:5_20:5) | Down |

**Table S4.** Results of differential metabolite screening between the control and the INH-ILI 14-day groups.

| Index | Compounds | Type |
|---|---|---|
| LIPID-N-0993 | PC (14:1_16:1) | Up |
| MEDP1933*117 | Cyclopentylglycine | Up |
| MEDP1974*117 | 1-Methylpiperidine-2-carboxylic acid | Up |
| MEDN0439 | N-Acetylthreonine | Up |
| LIPID-N-1044 | PC (15:0_22:6) | Up |
| LIPID-N-1403 | PI (15:1_16:1) | Up |
| LIPID-N-0458 | PG (16:1_20:4) | Up |
| LIPID-N-0533 | PI (20:4_16:1) | Up |
| MEDP1029 | Hispidol | Up |
| MEDP2255 | Chrysoeriol | Up |
| MEDP1274 | (4Z,7Z,10Z,13Z,16Z,19Z)-Eicosahexaenoate | Up |
| MEDP1288 | Chrysophanol | Up |
| MEDN1181 | 5-hydroxyindole acetaldehyde | Up |
| MEDP1393 | Carnitine C18:4 | Up |
| MEDN0776 | 5(S),15(S)-DiHETE | Up |
| MEDP2870 | Carnitine C22:5 | Up |
| MEDN0771 | 15-oxoETE | Up |
| MEDN1824 | 3-keto Petromyzonol | Up |
| LIPID-N-1171 | PG (16:1_16:2) | Up |
| LIPID-N-0982 | PC (12:0_16:1) | Up |
| LIPID-P-1957 | Cer (t18:1/40:2(2OH)) | Down |
| MEDN1880 | Hydroferulic acid | Down |
| LIPID-P-1020 | TG (24:0_18:1_18:2) | Down |
| MEDP1379 | Carnitine C21:3 | Down |
| MEDP1040 | N-(2-hydroxyethyl) stearamide | Down |
| LIPID-P-2339 | TG (20:0_18:2_18:2) | Down |

| Index | Compounds | Type |
| --- | --- | --- |
| LIPID-P-1008 | TG (18:1_18:2_22:0) | Down |
| MEDN1886 | L-3-Phenyllactic acid | Down |
| LIPID-P-2497 | Cer(d17:3/42:1(2OH)) | Down |
| MEDN1213 | 2-(4-hydroxyphenyl) propionate | Down |
| LIPID-P-2613 | Cer (d27:2/32:2(2OH)) | Down |
| LIPID-P-2622 | Cer (d28:3/31:1(2OH)) | Down |
| LIPID-P-2576 | Cer (d23:2/36:2(2OH)) | Down |
| LIPID-P-1126 | TG (24:1_18:2_18:2) | Down |
| LIPID-P-2551 | Cer (d19:3/40:1(2OH)) | Down |
| LIPID-P-2631 | Cer (d29:2/30:2(2OH)) | Down |
| LIPID-P-2604 | Cer (d26:3/33:1(2OH)) | Down |
| LIPID-P-1056 | TG (16:0_18:0_20:4) | Down |
| MEDN0485 | 1,6-di-O-phosphono-D-fructose | Down |
| LIPID-P-2578 | Cer (d23:3/36:1(2OH)) | Down |

**Table S5.** Results of differential metabolite screening between the control and the INH-ILI 28-day groups.

| Index | Compounds | Type |
|---|---|---|
| LIPID-N-0458 | PG (16:1_20:4) | Up |
| MEDN0792 | PGE1 | Up |
| MEDN1814 | Dihydrodaidzein | Up |
| MEDN1597 | Phosphatidylethanolamine lyso alkenyl 16:0 | Up |
| LIPID-N-0993 | PC (14:1_16:1) | Up |
| LIPID-N-1044 | PC (15:0_22:6) | Up |
| LIPID-N-0534 | PI (16:0_20:5) | Up |
| MEDN1491 | Equol | Up |
| MEDN1824 | 3-keto Petromyzonol | Up |
| MEDN1082 | 8-isoprostaglandin F1α | Up |
| LIPID-N-0533 | PI (20:4_16:1) | Up |
| MEDN0536 | estrone 3-sulfate | Up |
| LIPID-N-0056 | PGE2 | Up |
| MEDP2092 | 2,4-Quinolinediol | Up |
| MEDP2255 | Chrysoeriol | Up |
| MEDN2285 | Chrysin-7-O-glucuronide | Up |
| MEDP1029 | Hispidol | Up |
| LIPID-N-1171 | PG (16:1_16:2) | Up |
| MEDP1288 | Chrysophanol | Up |
| LIPID-N-0982 | PC (12:0_16:1) | Up |
| LIPID-P-1127 | TG (14:1_18:2_18:3) | Down |
| LIPID-P-1193 | TG (14:1_16:1_22:6) | Down |
| LIPID-P-1032 | TG (12:0_18:1_18:3) | Down |
| LIPID-P-2497 | Cer (d17:3/42:1(2OH)) | Down |
| LIPID-P-2607 | Cer (d27:1/30:1(2OH)) | Down |
| LIPID-P-2622 | Cer (d28:3/31:1(2OH)) | Down |

| Index | Compounds | Type |
|---|---|---|
| LIPID-P-2631 | Cer (d29:2/30:2(2OH)) | Down |
| LIPID-P-1232 | TG (14:0_20:5_22:6) | Down |
| LIPID-P-1239 | TG (14:0_22:6_22:6) | Down |
| LIPID-P-2495 | Cer (d17:2/42:1(2OH)) | Down |
| LIPID-P-2576 | Cer (d23:2/36:2(2OH)) | Down |
| LIPID-P-2613 | Cer (d27:2/32:2(2OH)) | Down |
| LIPID-P-2598 | Cer (d25:3/34:1(2OH)) | Down |
| LIPID-P-2274 | TG (12:0_16:1_22:6) | Down |
| LIPID-P-2575 | Cer (d23:2/36:1(2OH)) | Down |
| LIPID-P-1998 | Cer (t28:2/30:2(2OH)) | Down |
| LIPID-P-2637 | Cer (d30:3/29:0(2OH)) | Down |
| LIPID-P-2305 | TG (15:1_18:1_18:3) | Down |
| LIPID-P-1130 | TG (14:0_16:1_20:5) | Down |
| LIPID-P-2001 | Cer (t30:2/28:2(2OH)) | Down |

## References

[1] Reagan-Shaw S, Nihal M, Ahmad N. Dose translation from animal to human studies revisited. FASEB J. 2008; 22: 659-61.

[2] WHO. WHO consolidated guidelines on tuberculosis: module 4: treatment: drug-susceptible tuberculosis treatment. Accept 4 May 2022. https://iris.who.int/handle/10665/353829

[3] Li Y, Ren Q, Zhu L, Li Y, Li J, Zhang Y, et al. Involvement of methylation of microRNA-122, -125b and -106b in regulation of cyclin G1, CAT-1 and STAT3 target genes in isoniazid-induced liver injury. BMC Pharmacol Toxicol. 2018; 19: 11.

[4] Li Y, Li Y, Zheng G, Zhu L, Wang J, Mu S, et al. Cytochrome P450 1A1 and 1B1 promoter CpG island methylation regulates rat liver injury induced by isoniazid. Mol Med Rep. 2018; 17: 753-62.

[5] Huang L, Sun H, Sun L, Shi K, Chen Y, Ren X, et al. Rapid, label-free histopathological diagnosis of liver cancer based on Raman spectroscopy and deep learning. Nat Commun. 2023; 14: 48.

[6] Cheng WT, Liu MT, Liu HN, Lin SY. Micro-Raman spectroscopy used to identify and grade human skin pilomatrixoma. Microsc Res Tech. 2005; 68: 75-9.

[7] Stone N, Kendall C, Smith J, Crow P, Barr H. Raman spectroscopy for identification of epithelial cancers. Faraday Discuss. 2004; 126: 141-57; discussion 69-83.

[8] Huang Z, McWilliams A, Lui H, McLean DI, Lam S, Zeng H, et al. Near-infrared Raman spectroscopy for optical diagnosis of lung cancer. Int J Cancer. 2003; 107: 1047-52.

[9] Dukor RK. Vibrational spectroscopy in the detection of cancer. Handbook of vibrational spectroscopy. 2001.

[10] Jyothi Lakshmi R, Kartha VB, Murali Krishna C, JG RS, Ullas G, Uma Devi P, et al. Tissue Raman spectroscopy for the study of radiation damage: brain irradiation of mice. Radiat Res. 2002; 157: 175-82.

[11] Sivakumar S, Khatiwada CP, Sivasubramanian J, Raja B. FT-Raman study of deferoxamine and deferiprone exhibits potent amelioration of structural changes in the liver tissues of mice due to aluminum exposure. Spectrochim Acta A Mol Biomol Spectrosc. 2014; 118: 461-9.

[12] Wang H, Huang N, Zhao J, Lui H, Korbelik M, Zeng H, et al. Depth-resolved in vivo micro-Raman spectroscopy of a murine skin tumor model reveals cancer-specific spectral biomarkers. J Raman Spectrosc. 2011; 42: 160-6.

[13] Caspers PJ, Lucassen GW, Wolthuis R, Bruining HA, Puppels GJ. In vitro and in vivo Raman spectroscopy of human skin. Biospectroscopy. 1998; 4: S31-9.

[14] Demos SG, Vogel AJ, Gandjbakhche AH. Advances in optical spectroscopy and imaging of breast lesions. J Mammary Gland Biol Neoplasia. 2006; 11: 165-81.

[15] Yan J, Yu Y, Kang JW, Tam ZY, Xu S, Fong ELS, et al. Development of a classification model for non-alcoholic steatohepatitis (NASH) using confocal Raman micro-spectroscopy. J Biophotonics. 2017; 10: 1703-13.

[16] Gao S, Lin Y, Zheng M, Lin Y, Lin K, Xie S, et al. Label-free determination of liver cancer stages using surface-enhanced Raman scattering coupled with preferential adsorption of hydroxyapatite microspheres. Anal Methods. 2021; 13: 3885-93.

[17] Almond LM, Hutchings J, Lloyd G, Barr H, Shepherd N, Day J, et al. Endoscopic Raman spectroscopy enables objective diagnosis of dysplasia in barrett's esophagus. Gastrointest Endosc. 2014; 79: 37-45.

[18] Lau DP, Huang Z, Lui H, Anderson DW, Berean K, Morrison MD, et al. Raman spectroscopy for optical diagnosis in the larynx: preliminary findings. Lasers Surg Med. 2005; 37: 192-200.

[19] Liu M, Mu J, Wang M, Hu C, Ji J, Wen C, et al. Impacts of polypropylene microplastics on lipid profiles of mouse liver uncovered by lipidomics analysis and Raman spectroscopy. J Hazard Mater. 2023; 458: 131918.
[20] Gaggini MC, Navarro RS, Stefanini AR, Sano RS, Silveira L, Jr. Correlation between METAVIR scores and Raman spectroscopy in liver lesions induced by hepatitis C virus: a preliminary study. Lasers Med Sci. 2015; 30: 1347-55.
[21] Silveira L, Jr., Sathaiah S, Zangaro RA, Pacheco MT, Chavantes MC, Pasqualucci CA, et al. Correlation between near-infrared Raman spectroscopy and the histopathological analysis of atherosclerosis in human coronary arteries. Lasers Surg Med. 2002; 30: 290-7.
[22] Hanlon EB, Manoharan R, Koo TW, Shafer KE, Motz JT, Fitzmaurice M, et al. Prospects for in vivo Raman spectroscopy. Phys Med Biol. 2000; 45: R1-59.
